# Correlating gut microbial membership to brown bear health metrics

**DOI:** 10.1038/s41598-022-19527-4

**Published:** 2022-09-22

**Authors:** Sarah M. Trujillo, Erin A. McKenney, Grant V. Hilderbrand, Lindsey S. Mangipane, Matthew C. Rogers, Kyle Joly, David D. Gustine, Joy A. Erlenbach, Buck A. Mangipane, Diana J. R. Lafferty

**Affiliations:** 1 Wildlife Ecology and Conservation Science Lab, Department of Biology, Northern Michigan University, Marquette, MI 49855 USA; 2 Department of Applied Ecology, North Carolina State University, Raleigh, NC 27607 USA; 3 Natural Resources Team, National Park Service, Anchorage, AK 99501 USA; 4 Marine Mammals Management, U.S. Fish and Wildlife Service, Anchorage, AK 99503 USA; 5 National Oceanic and Atmospheric Administration, National Marine Fisheries Service, Juneau, AK 99801 USA; 6 Gates of the Arctic National Park and Preserve, National Park Service, Fairbanks, AK 99709 USA; 7 Kodiak National Wildlife Refuge, U.S. Fish and Wildlife Service, Kodiak, AK 99615 USA; 8 Lake Clark National Park and Preserve, National Park Service, Anchorage, AK 99501 USA

**Keywords:** Microbiome, Ecology, Conservation biology

## Abstract

The internal mechanisms responsible for modulating physiological condition, particularly those performed by the gut microbiome (GMB), remain under-explored in wildlife. However, as latitudinal and seasonal shifts in resource availability occur, the myriad micro-ecosystem services facilitated by the GMB may be especially important to wildlife health and resilience. Here, we use brown bears (*Ursus arctos*) as an ecological model to quantify the relationship between wildlife body condition metrics that are commonly used to assess individual and population-level health and GMB community composition and structure. To achieve these aims, we subsampled brown bear fecal samples collected during United States National Park Service research activities at three National Parks and Preserves (Katmai, Lake Clark, and Gates of the Arctic) and extracted microbial DNA for 16S rRNA amplicon sequencing and microbial taxonomic classification. We analyzed GMB communities using alpha diversity indices, subsequently using Spearman’s correlation analysis to examine relationships between alpha diversity and brown bear health metrics. We found no differences in GMB composition among bears with differing body conditions, nor any correlations between alpha diversity and body condition. Our results indicate that GMB composition reflects diverse foraging strategies while allowing brown bears to achieve similar body condition outcomes.

## Introduction

The niche variation hypothesis (NVH^1^) predicts that species with broader niches will exhibit greater among-individual diet variation or individual specialization and that individual variation in dietary niche should confer an adaptive advantage. Researchers have used a range of methods to test the NVH, from evaluating diet variation to measuring physiological conditions related to fitness. For example, Bolnick et al.^2^ found that generalist species (e.g., three-spine stickleback [*Gasterosteus aculeatus*], Eurasian perch [*Perca fluviatilis*], Anolis lizards, intertidal gastropods, and neotropical frogs) do in fact display greater among-individual dietary niche variation compared to species with more specialized feeding strategies. While many generalist consumers exhibit dietary plasticity and variation^3^, it is unclear if these adaptations increase species resiliency when shifts in resource availability occur. However, at the ecosystem-level, when the range of natural variation (i.e., biodiversity)^4^ is reduced, so is resilience in ecosystem functionality^5^. Furthermore, in many systems, biodiversity is driven in part by multitrophic interactions dependent on the width of generalist consumers’ dietary niches^6^. As such, the successful management of ecosystems may depend on effectively protecting the resources that represent the full range of dietary niches represented within a population of generalist consumers. In addition, Lafferty et al.^7^ and Mangipane et al.^8,9^ found that individual brown bears (*Urus arctos*) achieve similar body condition independent of individual variation in dietary niches, suggesting that the diets consumed by different individuals can confer similar physiological benefits. However, the internal mechanisms responsible for modulating physiological condition, particularly those performed by the gut microbiome (GMB), remain under-explored in wildlife despite evidence that the GMB plays an important role in host resiliency to global environmental change^10^. The myriad micro-ecosystem services facilitated by the GMB^11^ may be especially important as generalist consumers respond to changes in resource availability.

GMBs co-evolved to complement host physiology and metabolism^12,13^; and the GMB’s collective genetic breadth and capacity for faster evolution further augments the host’s dietary plasticity. As such, the GMB can serve the host as a buffer against environmental perturbations and shifts in resource availability by promoting nutritional efficiency^14^ and adjusting fat storage^13^. While the GMB is an integral link between host diet and body condition, much of our knowledge regarding patterns of GMB community membership and host physiological condition comes from studies in humans and model organisms. For example, studies in obese humans (*Homo sapiens*) and mice (*Peromyscus* spp.) reveal patterns in the relative abundance of bacteria phyla such as Bacteroidetes and Firmicutes that are associated with an increased capacity to harvest energy from the diet^15^. Obesity in humans is also correlated to GMB dysbiosis (i.e., imbalance or breakdown of community structure) that can lead to an array of diseases detrimental to host health^16^. Yet, in wildlife species, particularly species that hibernate or go into torpor, fat accumulation is critical to survival and is not associated with negative health effects. In fact, in contrast to humans, brown bears remain metabolically healthy during hyperphagia (i.e., rapid weight gain)^17^; when bears consume ~ 20,000 cal a day^18^. Given that GMB communities are shaped in part by host phylogeny^19^ and diet^20^, it is important to investigate species-specific patterns in GMB community composition to better understand how the GMB correlates to body condition.

Brown bears are generalist consumers that forage across trophic levels based on food availability, nutritional needs, and competition, and thus drive widespread ecosystem effects^21–23^. As such brown bears provide an ecologically relevant model for quantifying the relationship between wildlife body condition metrics that are commonly used to assess individual and population-level health and GMB community composition and structure. Here, we aimed to (1) characterize brown bear GMB community composition associated with body condition as measured by percent body fat, lean mass, fat mass, and net mass, (2) examine differences between the relative abundance of specific GMB bacterial taxa, alpha diversity among bears with differing body conditions, and beta diversity, and (3) assess correlations between specific GMB bacterial phyla, genera, and alpha diversity among bears with differing body conditions. We hypothesized that brown bears with different body conditions would harbor unique GMB communities. Specifically, we predicted that differences in phylum and genus-level bacterial relative abundances and GMB diversity indices would exist between bears with above median and below median body condition. We further hypothesized that body condition would correlate to GMB diversity because more diverse microbial communities can facilitate greater nutrient absorption and energy storage^24^ while phyla and genera relative abundance would correlate to body condition. To test these hypotheses, we measured percent body fat, net mass, lean body mass, and fat mass, and used 16S rRNA amplicon sequencing to characterize GMB communities from three populations of brown bears across Alaska.

## Results

### Community composition

**Figure 1:**
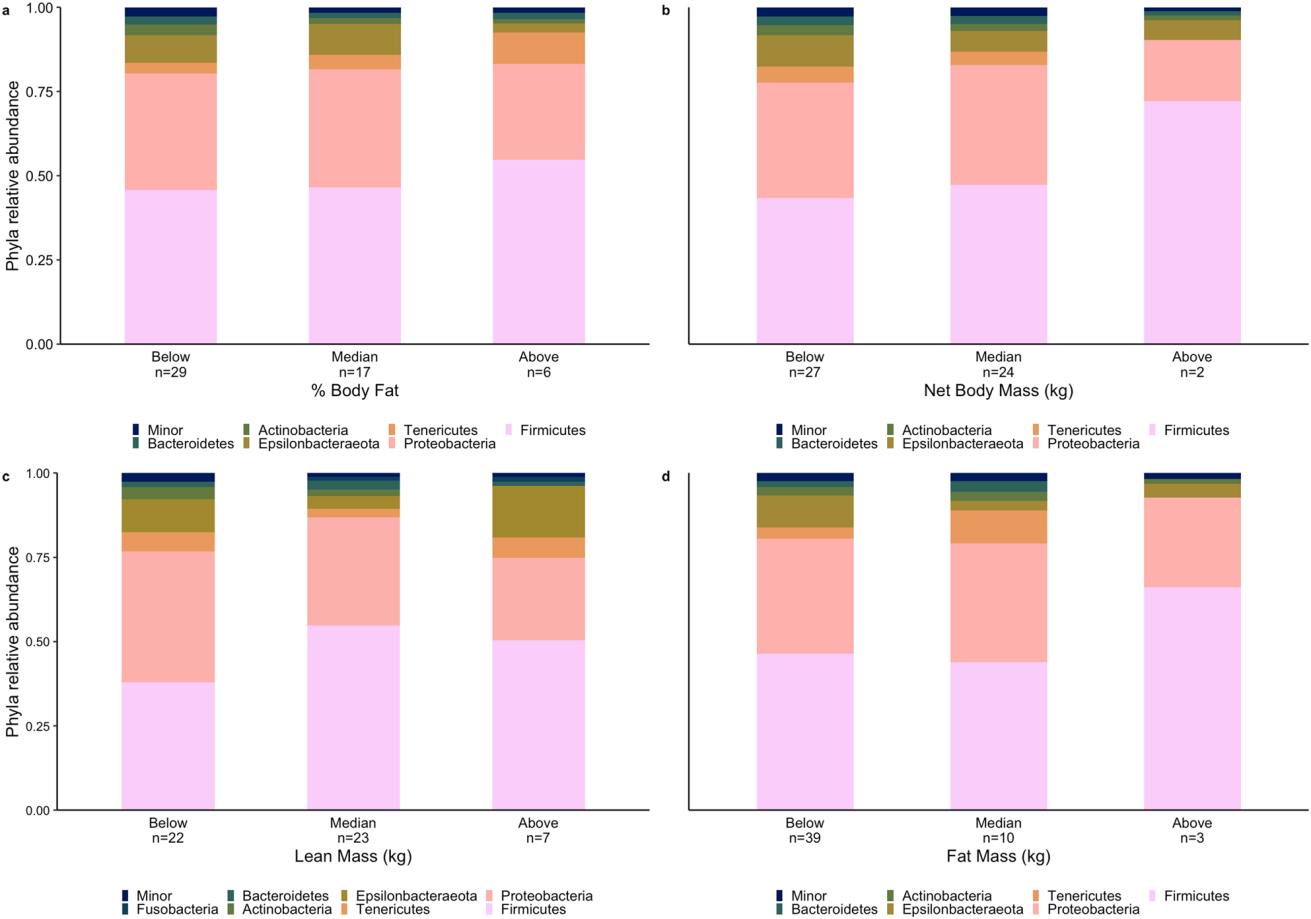
Relative abundance of the major GMB bacterial phyla identified across (**a**) percent body fat; (**b**) net body mass; (**c**) lean mass; and (**d**) fat mass in Alaskan brown bear (*Ursus arctos*). We include all major taxa occurring at ≥ 1% relative abundance; “minor” taxa are those occurring at < 1% relative abundance. Body metrics categories were created using median splitting.

We identified seven major bacterial phyla (relative abundance ≥ 1%) among brown bears sampled across body metric categories, with five phyla shared among all groups (Actinobacteria, Bacteroidetes, Epsilonbacteraeota, Firmicutes, Proteobacteria). Firmicutes and Proteobacteria were the dominant bacterial phyla in brown bear GMB communities, with the relative abundance of Firmicutes at 49.46% (± 32.44 SD) and the relative abundance of Proteobacteria at 31.18% (± 27.20 SD; Supplement, Table S1). Tenericutes were detected in most brown bear body condition groups, excluding above median net mass and above median fat mass. Fusobacteria were only detected in abundances > 1% in brown bears with above median lean mass (Fig. 1; Supplement, Figs. S1–S4).

We identified 14 major bacterial genera (relative abundance ≥ 1%) and 4 additional major taxa not identified to the genus level across percent body fat median-split categories (i.e., below median, median, and above median) (Supplement, Fig. S5). Each body fat category was associated with 8 major bacterial taxa. *Escherichia-Shigella* dominated the GMBs of bears with below median percent body fat. *Bacteroides* and *Paenalcaligenes* were unique to bears with below median percent body fat. *Actinobacillus* were only identified in bears with median percent body fat, while minor taxa (relative abundance < 1%) comprised 19% of the total GMB. *Pseudomonas* were unique to bears with above median percent body fat, while *Streptococcus* dominated those samples.

Across net body mass median-split categories, we identified 15 major genera and 4 additional major taxa not identified to the genus level (Supplement, Fig. S6). Seven major taxa were shared across all net body mass categories. Bears with below median net body mass had one unique genus (*Paenalcaligenes*) and were dominated by *Escherichia-Shigella*. *Actinobacillus*, *Cellulosilyticum*, *Edwardsiella*, and *Terrisporobacter* were all unique to bears with median net body mass. Median net body mass GMBs were dominated by minor taxa. *Pseudomonas* were only identified in bears with above median net body masses. The GMBs of bears with above median net body mass were dominated by taxon only identified to the order Lactobacillales.

We identified 15 major genera and 4 major taxa unidentified at the genus level within the lean mass categories (Supplement, Fig. S7). Like bears with below net body mass, bears with below median lean mass had one unique genus (*Paenalcaligenes*) and were dominated by *Escherichia-Shigella*. The GMBs of bears with median lean mass were dominated by minor taxa and had one unique genus (*Edwardsiella*). *Actinobacillus* and *Cellulosilyticum* were only identified in bears with above median lean mass. The GMBs of bears with above median net body mass were dominated by *Escherichia-Shigella*.

We identified 17 major genera (relative abundance ≥ 1%) and 5 major taxa unidentified at the genus level among fat mass categories (Supplement, Fig. S8). *Ureaplasma* were only detected in bears with below median fat mass measurements. GMBs within the below median fat mass category were dominated by *Escherichia-Shigella*. *Actinobacillus*, *Allorhizobium-Neorhizobium-Pararhizobium-Rhizobium, Bibersteinia, Cellulosilyticum*, *Lactobacillus*, *Mycoplasma*, and *Ursidibacter* were all unique to bears with median fat mass. The GMBs of bears with median fat mass were dominated by minor taxa. The GMBs of bears with above median fat mass were dominated by a taxa only identified to the order Lactobacillales and had 1 unique genus (*Pseudomonas*).

We detected *Ursidibacter arcticus* in 8 bears across all parks with relative abundance within individuals ranging from 0.02 to 15.73%.

### GMB differences among body conditions

The average relative abundance of Actinobacteria was highest in bears with below median percent body fat (3.1% ± 5.8 SD), below median net mass (3.0% ± 5.9 SD), below median lean mass (3.7% ± 6.4 SD), and median fat mass (2.7% ± 3.7 SD; Supplement, Table S2). Actinobacteria were not detected at ≥ 1% in bears with above median fat mass. Bacteroidetes were most abundant in bears with below median percent body fat (2.3% ± 5.5 SD), below median net mass (2.5% ± 5.8 SD), median lean mass (2.6% ± 6.7 SD), and median fat mass (3.2% ± 4.9 SD; Supplement, Table S2). Bacteroidetes were not present at ≥ 1% in bears with above median fat mass. Epsilonbacteraeota were highest in the GMBs of bears with median percent body fat (9.1% 18.8 SD), below median net mass (9.3% ± 15.4 SD), above median lean mass (15.4% ± 27.1 SD), and below median fat mass (9.5% ± 17.4 SD; Supplement, Table S2). The average relative abundance of Firmicutes in GMBs was highest in bears with above median body fat (54.7% ± 22.3 SD), above median net mass (72.1% ± 26.2 SD), median lean mass (54.8% ± 27.4 SD), and above median fat mass (66.2% ± 21.2 SD; Supplement, Table S2). The average relative abundance of Proteobacteria was highest in the GMBs of bears with median percent body fat (35.0% ± 28.8 SD), median net mass (35.6% ± 28.4 SD), below median lean mass (38.8% ± 29.0 SD), and median fat mass (35.2% ± 27.5 SD; Supplement, Table S2). Tenericutes average relative abundance was highest in bears with above median percent body fat (9.4% ± 22.9 SD), below median net mass (4.7% ± 12.1 SD), above median lean mass (5.9% ± 9.4 SD), and median fat mass (9.8% ± 18.6 SD; Supplement, Table S2). Tenericutes were not detected in abundances ≥ 1% in bears with above median net mass or above median fat mass. There were no significant differences in the average abundance of brown bear major GMB bacterial phyla between below median, median, and above median categories of each brown bear health metric (Supplement, Tables S3, S4).

The average relative abundance of *Escherichia-Shigella* was highest in bears with below median percent body fat (24.5% ± 29.5 SD), below median net mass (25.7% ± 28.9 SD), below median lean mass (28.9% ± 30.6 SD), and below median fat mass (24.0% ± 28.5 SD; Supplement, Table S5). *Streptococcus* was most abundant in bears with above median percent body fat (17.5% ± 23.6 SD), median net mass (14.5% ± 23.2 SD), median lean mass (15.1% ± 23.6 SD), and above median fat mass (18.2% ± 27.2 SD; Supplement, Table S5). *Turicibacter* was highest in the GMBs of bears with below median percent body fat (16.6% ± 23.7 SD), above median net mass (26.6% ± 37.5 SD), median lean mass (17.5% ± 24.0 SD), and median fat mass (86.4% ± 12.1 SD; Supplement, Table S5). The average relative abundance of *Clostridium *sensu stricto* 1* in GMBs was highest in bears with median body fat (10.2% ± 17.4 SD), below median net mass (8.9% ± 15.1 SD), median lean mass (9.4% ± 14.6 SD), and below median fat mass (9.9% ± 15.8 SD; Supplement, Table S5). *Clostridium *sensu stricto* 1* was not detected at ≥ 1% in bears with above median fat mass. *Helicobacter* were most abundant in bears with median percent body fat (8.6% ± 15.5 SD), below median net mass (8.9% ± 14.5 SD), above median lean mass (15.3% ± 27.1 SD), and below median fat mass (9.1% ± 16.9 SD; Supplement, Table S5). There were no significant differences in the average abundance of brown bear major GMB bacterial genera between below median, median, and above median categories of each brown bear health metric (Supplement, Tables S6, S7).

The average Faith’s PD value was highest in bears with above median body fat (12.6 ± 6.5 SD), median net mass (11.0 ± 7.3 SD), below median lean mass (11.6 ± 8.8 SD), and median fat mass (13.8 ± 9.2 SD) (Supplement, Table S8). The average Shannon diversity value was highest in bears with median body metrics (Supplement, Table S8). Inverse Simpson index was lowest (more diverse) in bears with above median body metrics (Supplement, Table S8). Despite these trends, there were no significant differences in alpha diversity indices among bears with different body conditions (Supplement, Fig. S9, Table S9).

GMB beta diversity did not differ significantly among brown bears of varying body conditions (Supplement, Table S10). The dominant lineages of just a few bear GMBs with below median and median body conditions appear to drive the majority of the weighted UniFrac variation (Supplement, Fig. S10), and unweighted UniFrac distances suggest that most bacterial taxa are present across samples (Supplement, Fig. S11).

We found a total of 31 ASVs that were differentially represented between body condition measurements (Supplement, Table S11). In bears with median percent body fat, we identified 1 differentially abundant ASV belonging to a minor phylum. In bears with above median percent body fat, we identified a differentially abundant bacteria belonging to Firmicutes. In net mass body condition categories, we identified 2 differentially abundant ASVs in bears with median net mass (1 Firmicutes, 1 Proteobacteria) and 1 differentially abundant ASV belonging to a minor genus within Actinobacteria in bears with above median net mass. Between lean mass categories, we found 3 differentially abundant ASVs (2 Actinobacteria, 1 Bacteroidetes) in bears with below median lean mass, 2 differentially abundant ASVS (1 Epsilonbacteraeota, 1 Firmicutes) in bears with median lean mass, and 2 ASVs (1 Firmicutes, 1 Proteobacteria) in bears with above median lean mass categories. Between fat mass categories, we identified 15 ASVs (1 Actinobacteria, 8 Firmicutes, 5 minor phyla) that were differentially represented within bears with median fat mass and 4 ASVs (1 Actinobacteria, 1 Proteobacteria, 2 minor phyla) within bears with above median fat mass.

### Correlation between the GMB and body condition

**Figure 2:**
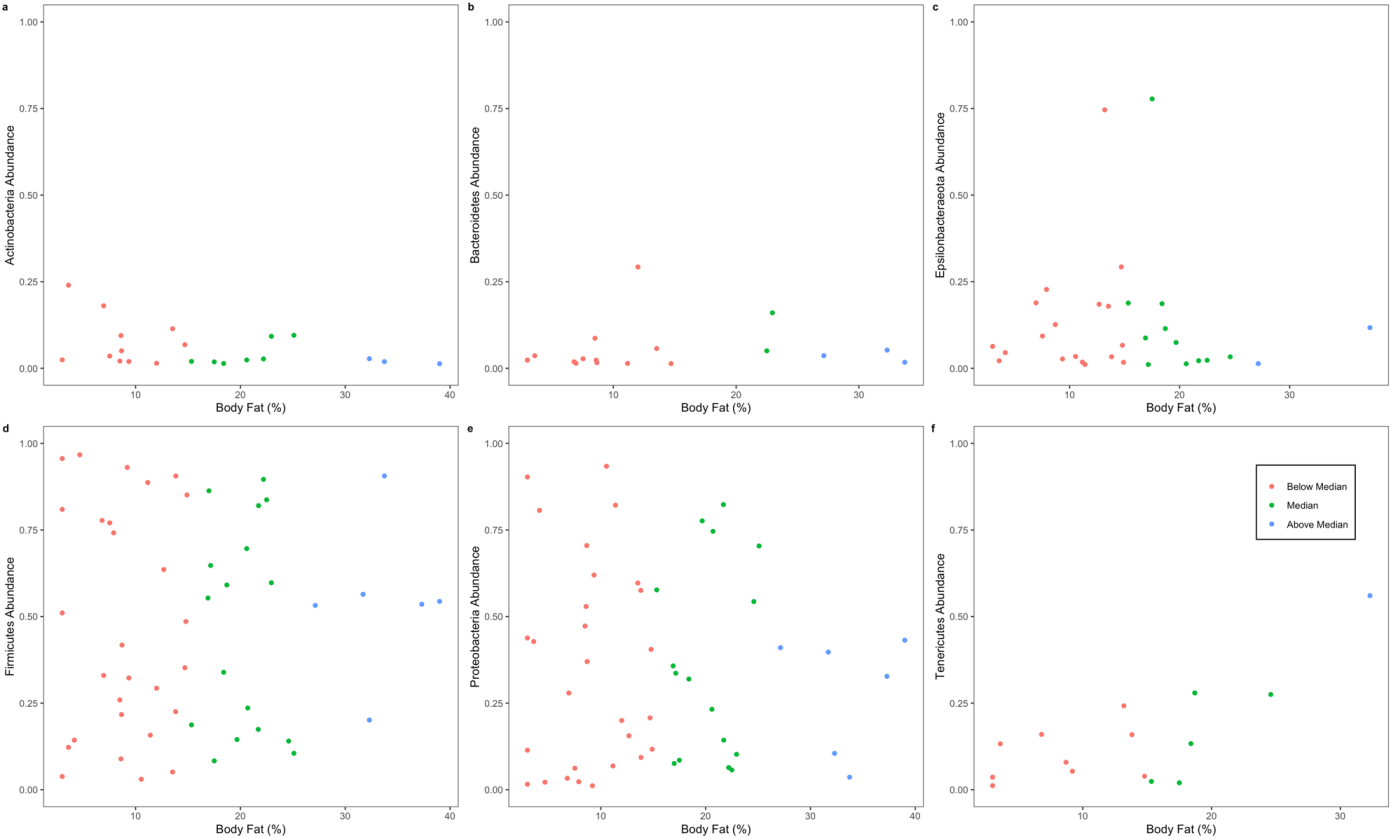
The relative abundance of major phyla (≥ 1%) in brown bear (*Ursus arctos*) gut microbiomes across individuals with different percent body fat.

None of the body condition measurements correlated to the relative abundance of any major phyla or dominant genera (p > 0.05; Fig. 2; Supplement, Table S12). Furthermore, none of the body condition measurements correlated to alpha diversity (p > 0.05; Supplement, Table S12).

## Discussion

Firmicutes dominating GMBs of all bears is consistent with research indicating Firmicutes are essential to the breakdown of complex plant carbohydrates and weight gain^25^. Within Firmicutes, the order Lactobacillales may dominate the GMBs of bears with above median body mass because they are facilitating efficient energy production of lactic acid. For example, burning lactic acid in addition to carbohydrates would allow bears to produce more energy than solely relying on carbohydrates for fuel^26^.

The GMBs of brown bears with poorer body condition were dominated by *E-Shigella*, which are opportunistic and potentially pathogenic. This is consistent with the possibility that these bears may lack sufficient fat or body mass to support fully functional immune systems^27^. Furthermore, *Paenalcaligenes* were only detected in the GMBs of bears with below median body conditions. *Paenalcaligene* are associated with cognitive impairment in humans and cause both cognitive impairment and colitis in mice^28^. In addition, *Ureaplasma* is associated with infection of human urogenital tracts^29^ and may also be an indication of a compromised immune system in bears with below median fat mass. However, it is possible that *Ureaplasma* are assisting in the essential urea cycling that occurs in the gut during hibernation^30^ or that bears with low body condition are not consuming enough food to support their microbiome, forcing their GMBs to shift toward alternate resources such as urea to support ATP synthesis.

To our knowledge, we report the first detection of *Ursidibacter arcticus* in a wild brown bear population. *Ursidibacter arcticus* was first described by Johanne Hansen et al.^31^ and is thought to have co-evolved and radiated with the Ursidae family.

While larger bears on average had a higher relative abundance of Firmicutes and lower abundances of Actinobacteria, consistent with the obese phenotype in rodents^32^, there were no significant differences in the relative abundance of either Firmicutes or Actinobacteria relative to body condition. While there was no correlation, the results of our genus-level analyses suggest that different taxa are adapted to specific host physiological conditions associated with different health metrics. Specifically, while *Escherichia-Shigella, Streptococcus, Clostridium *sensu*-stricto 1,* and *Helicobacter* have all been previously characterized as broadly “opportunistic” or “potentially pathogenic”^27,33–35^, the emerging patterns of the relative abundance of each taxon with a distinct set of host health metrics in our study suggests nuanced niche differentiation in response to host physiology. These results pave the way for future studies to investigate which specific physiological states, mechanisms, and pathways (e.g., host metabolic processes or immune and inflammatory status) might contribute to gut microbial regulation.

GMB alpha diversity was generally higher in larger bears, yet there were no significant differences in any alpha diversity indices relative to body condition categories. Assuming that bears with higher body fat are eating more food, this would support that their GMBs have more available niche space. However, recent studies demonstrate that brown bears can achieve similar body condition outcomes with variable foraging strategies^7–9^. Therefore, it is likely that bears with higher percent body fat, net mass, and fat mass still vary in the amounts of meat and fiber consumed per individual. While high fiber diets are associated with higher GMB diversity^36^, patterns of alpha diversity in brown bear GMBs may be masked by individual variation in dietary niches. Furthermore, because the GMB plays an essential role in a host’s ability to extract nutrients from their diet^37^ and nutrient quality is positively correlated with brown bear body condition measurements^38^, our findings may further support the NVH in that GMB composition reflects diverse foraging strategies while allowing brown bears to achieve similar body condition outcomes.

Based on correlation analyses we did not identify any significant relationships between body condition and any major phyla. The Firmicutes to Bacteroidetes ratio prevalent in obese mice^39^ was not correlated to above median body condition metrics in brown bears. However, previous research suggests that large brown bears and obese model organisms (e.g. mice, humans) differ metabolically^40^. Further, patterns of GMB community composition differ among host species^19^. Although calculating bacterial phyla relative abundance is an important part of assessing community composition, some studies suggest that a more robust understanding of the relationship between a host and their GMB requires microbial identification to the species level^41^. It is also possible that the contributions of minor taxa to brown bear physiological condition are underestimated. For example, keystone species have disproportionately large effects on their ecosystem relative to their abundance^42^.

Lack of correlation between brown bear body condition and their GMB alpha diversity suggests that the relationship between the GMB and body condition may be more complex than we were able to describe in this study and more nuanced than can be captured with a single summary statistic. In contrast, Amato et al.^43^ found that host phylogeny can have a greater influence over GMB alpha diversity than dietary niche in primates. Therefore, it is possible that brown bear phylogeny may be a better predictor of GMB alpha diversity than diet.

In congruence with the NVH, brown bears exhibit the ability to achieve similar body conditions across substantial range of among-individual dietary niche variation^7–9^. In a previous study, we characterized brown bear GMB bacterial community composition associated with location, season, and reproductive condition with taxa relative abundance^44^. We found that brown bear GMBs vary in membership and overall composition, complementary to the nutritional landscape of each location and that GMB composition presents similarly high levels of among-individual variation as diet. Further, while food resource availability and average bear size differed by location^45^, we did not consider any location as more or less “favorable” because all brown bears sampled were considered “healthy” (i.e., normal lean mass and body size)^45^. While assessing the relationship between an individual brown bear’s body metrics and GMB community composition may identify health implications associated with major taxa, brown bear body condition and GMB health may better be assessed by multiple taxonomic signatures. Instead, assessing inter-individual variation within a population may help managers identify shifts in GMB composition that impact the ability for brown bears to process myriad food resources, which enables brown bears to successfully occupy diverse landscapes. Managing for diverse GMBs may allow managers to foster bear populations that consist of individuals with a broader ability to derive nutrients from the variety of resources that are available at a given time (i.e., increased dietary plasticity facilitated by the GMB). For example, balancing the number of natural resources humans take (i.e., fisheries) with the provision of long term needs for brown bear populations would ensure the continuing availability of important food sources and GMB inputs for bears. Further, managing for diverse GMBs may include managing for “healthy” GMBs by not allowing bears to obtain human foods that might alter GMBs and negatively impact health. Managing to protect the full range of diversity represented within a population, including among-individual GMB diversity, may be beneficial to ensure brown bear populations are resilient when confronted with environmental change.

## Materials and methods

### Study area

**Figure 3:**
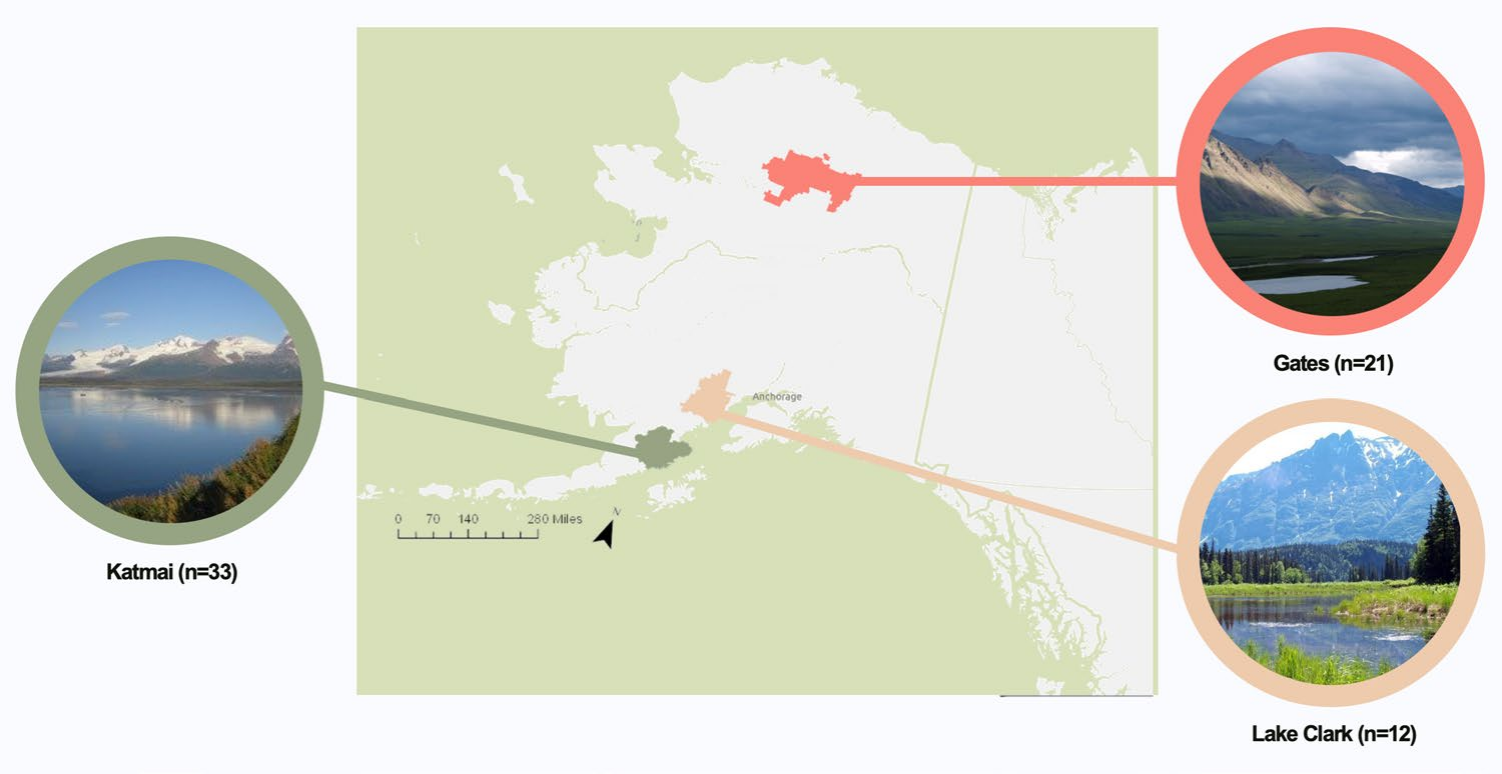
Map of study areas: Katmai National Park and Preserve (Katmai; n = 33), Lake Clark National Park and Preserve (Lake Clark; n = 12), and Gates of the Arctic National Park and Preserve (Gates; n = 21). Photos of parks by National Park Service. Map created using R (version 4.0.2.; R Core Team 2020), RStudio (version 1.3.1056; RStudio Team 2020), *sf* (R, version .0-7), *tmap* (R, version 3.3-3), and *spData* (R, version 2.0.1). Park boundary shapefile from National Park Service (https://irma.nps.gov/DataStore/).

Our study area encompassed regions of Katmai National Park and Preserve (Katmai), Lake Clark National Park and Preserve (Lake Clark), and Gates of the Arctic National Park and Preserve (Gates; Fig. 3). Katmai is located in southern Alaska on the Alaska Peninsula. Within Katmai, our study area included a section of the eastern Aleutian Range as well as coastal, intertidal, and island areas. Key food resources used by brown bears in Katmai include fish (e.g., salmon [*Oncorhynchus* spp.]), salt marsh vegetation (e.g., *Carex* spp. and *Plantago maritima*), berries and herbaceous vegetation^45^. Some Katmai bears were observed consuming unique food sources such as marine mammals (e.g., sea otter [*Enhydra lutris*], harbor seal [*Phoca vitulina*]), marine invertebrates, and flounder (*Platichthys stellatus*). Lake Clark is a historically glaciated ecosystem located in southcentral Alaska, between the Alaska and Aleutian Mountain Ranges. Our Lake Clark study area is characterized by subalpine tundra, spruce (*Picea* spp.) forest, and riparian zones within the Chigmit Mountains that divide the western interior region from the coastal region of the park^8^. Based on GPS-collar data^8^, Lake Clark study animals did not leave the western interior region and, consequently, did not have access to coastal resources. Instead, the Lake Clark study bears relied on herbaceous vegetation and berries, moose (*Alces alces*), caribou (*Rangifer tarandus*), Dall’s sheep (*Ovis dalli*), small mammals, and salmon^8,45^. Gates is located in northern Alaska above the Arctic Circle. The study area included a region on the south side of the Brooks Mountain Range characterized by tundra, spruce forest, and riparian zones^46^. Primary foods for brown bears in Gates are more limited than in southern systems due to a shorter growing season and low ungulate density^47^. However, Gates bears are known to consume small mammals (e.g., Arctic ground squirrels [*Urocitellus parryii*]), large mammals (i.e., moose, caribou, and Dall’s sheep), seasonal herbaceous vegetation and berries, and limited, seasonal salmon^8,45^.

### Body metric data collection and fecal sampling

We obtained 66 fecal samples from Katmai (n = 33), Lake Clark (n = 12), and Gates (n = 21). United States National Park Service (NPS) collected samples during population monitoring from 2015 to 2017. Bears were anesthetized via darting from a helicopter using tiletamine hydrochloride and zolazepam hydrochloride (Telazol^®^, Fort Dodge Laboratories, Fort Dodge, IA, USA) according to Taylor et al.^48^. Bears were weighed using an electronic load cell ($\pm$ 0.5 kg; MSI-7200; Measurement Systems International, Seattle, WA, USA) and body composition (body fat, lean mass, and fat mass) was determined by bioelectric impedance analyses (RJL Systems, Clinton Township, MI, USA)^49,50^. Individuals with body fat percentages below 3% (n = 4) were likely the result of measurement errors. We rounded body fat values up to 3% for these individuals according to Mangipane et al.^9^. Study methods have been followed in accordance with all guidelines and capture and handling procedures were approved by the Institutional Animal Care and Use Committees of the National Park Service (AKR_KATM_Hilderbrand_Brown-Bear_2014, AKR_LACL_Mangipane_BrownBear_2014, AKR_GAAR_Gustine_GrizzlyBear_2014) and the U.S. Geological Survey, Alaska Science Center (2014-01, 2015-04, 2015-06). Biological samples (e.g., feces, hair, blood) collected during these research activities were archived along with the physiological data for each animal (e.g., reproductive condition, age, net mass, lean body mass, fat mass, percentage body fat; Supplement, Table S13) as well as environmental data associated with capture locations (e.g., elevation, land cover/habitat). Fecal samples were immediately stored in 95% ethanol upon collection then in a − 20 °C freezer for the long term.

### Methods

#### Laboratory methods

We used DNeasy PowerSoil Kits (QIAGEN) to extract microbial DNA from brown bear fecal samples. Though we followed the manufacturer’s protocol, we included an additional heat incubation period to further break down proteins in the feces and a second elution step to increase DNA yields^51^. We used a NanoDrop 2000c (ThermoFischer Scientific, MA, USA) to quantify DNA yields and then stored the DNA extracts at − 80 °C. We sent standardized DNA aliquots to Argonne National Laboratory (Lemont, IL, USA) for amplicon library preparation and paired-end multiplexed sequencing of the 16S rRNA hypervariable v4 gene region. As a quality control measure, Argonne National Laboratory includes negative PCR controls in every plate amplified and proceeds with sequencing only if negative controls are uncontaminated/clean.

#### Bioinformatics analysis

We imported the microbial sequence reads received from Argonne National Laboratory into Quantitative Insights Into Microbial Ecology (QIIME2, version 2019.4). Using DADA2 QIIME2 plugin^52^, we joined raw sequences, quality-filtered, demultiplexed, and called the amplicon sequence variants (ASVs) for analysis. We filtered the sequences to remove chloroplasts, mitochondria, Archaea, and all microbial sequences unidentified below the kingdom level. Next, we classified microbial taxa to the genus level using the SILVA 99 database (version 132^53^).

Due to unequal ASV count data that can lead to subsequent biases^54^, we normalized samples at a C_min_ depth of 4087 for 253,394 total sequences (11.41% of the original input), retaining 62 samples. Normalization was accomplished using scaling with ranked subsampling (SRS^55^), a method that retains the same relative frequencies of all species. First, SRS divides all ASVs by a scaling factor so that the sum of scaled counts equals a selected total number of counts (C_min_). SRS then ranks ASVs by converting non-integer counts into integers to minimize subsampling error^55^.

#### Statistical analysis

##### Community composition

We used R (version 4.0.2.; R Core Team 2020) and Rstudio (version 1.3.1056; RStudio Team 2020) for all statistical analyses and visualizations. Data were imported into R using *qiime2R* (R, version 0.99.34) then converted to *phyloseq* (R, version 1.32.0) objects. We used median splitting to create three categories (i.e., below median, median, and above median) for all body condition measurements (i.e., percent body fat, net mass, lean body mass, and fat mass; Table 1). We identified major GMB bacterial phyla (relative abundance ≥ 1%) and calculated relative abundancies of major taxa to visualize brown bear GMB communities associated with each brown bear health metric using categorization based on median splitting. Major taxa are defined by the 1% threshold based on definitions described in previous studies in order to identify the roles and contribution of all microbial taxa^56,57^.

**Table 1:** Alaskan brown bear (*Ursus arctos*) body condition categories.

	Below median	Median	Above median
Body fat (%)	3.0–15.0	15.1–27.0	27.1–39.0
Net body mass (kg)	76.8–152.0	152.1–228.0	228.1–303.0
Lean mass (kg)	72.6–115.0	115.1–157.0	157.1–199.0
Fat mass (kg)	1.0–38.3	38.4–75.6	75.6–113.0

Body metric categories were created using median splitting.

##### GMB differences among body conditions

After calculating relative abundancies of major bacterial taxa associated with each brown bear body condition metric category, we used combination of one-way analysis of variance (ANOVA) and non-parametric Kruskal–Wallis rank sum tests with Bonferroni corrections to test for significant differences between mean major bacterial phyla abundance in different body condition categories. We then used we used Linear discrimination analysis effect size (LEfSe) with the Galaxy online tool (https://huttenhower.sph.harvard.edu/galaxy) to identify any ASVs that were significantly enriched between body condition categories. We designated a logarithmic linear discriminate analysis (LDA) score of 2.0 as the cut-off for LEfSe analysis^58^.

We quantified the alpha diversity of GMB bacterial communities using microbiome (R, version 1.10.0) and picante (R, version 1.8.2). We used Shannon’s diversity index to quantify GMB bacterial community richness and evenness^59^, and we used Faith’s Phylogenetic Diversity (Faith’s PD^60^) to qualitatively assess GMB bacterial community richness and phylogenetic relationships. Additionally, we used inverse Simpson’s diversity index^61^ to quantify both the richness and evenness of microbial communities while incorporating phylogenetic relationships. We then used ANOVA tests to test for significant differences between mean alpha diversity indices across different body conditions and Tukey’s honestly significant difference post hoc tests to identify which body condition categories were significantly different from one another.

We compared pairwise GMB beta diversity using weighted and unweighted UniFrac distance matrices^62^, with weighted-UniFrac incorporating the relative abundance of taxa shared between samples and unweighted-UniFrac reflecting species presence/absence. We used multivariate analysis of variance, W^*^_d_ test^63^, to test differences amongbody conditions and the T^2^_*w*_ test^64^ with Bonferroni’s correction post hoc for significant factors.

##### Correlation between the GMB and body condition

We then used Spearman’s correlation analysis to examine the association between major phyla, dominant genera, and each body condition metric. Additionally, we used Spearman’s correlation analyses to examine the relationship between GMB bacterial alpha diversity indices and each body metric.

## Supplementary Information

Supplementary Information.

## Data Availability

The datasets generated during and/or analyzed during the current study (i.e., phyloseq-R objects from imported qiime2 artifacts, demultiplexed EMP-paired end sequences from Argonne National laboratory, and R code) are available in the Zenodo repository, https://zenodo.org/record/5759055#.YbuUqvHMLVY.

## References

[1] Van Valen L (1965). Morphological variation and width of ecological niche. Am. Nat..

[2] Bolnick DI, Svanbäck R, Araújo MS, Persson L (2007). Comparative support for the niche variation hypothesis that more generalized populations also are more heterogeneous. PNAS.

[3] Bearhop S, Adams CE, Waldron S, Fuller RA, Macleod H (2004). Determining trophic niche width: A novel approach using stable isotope analysis. J. Anim. Ecol..

[4] Hooper DU (2005). Effects of biodiversity on ecosystem functioning: A consensus of current knowledge. Ecol. Monogr..

[5] Roederer JG, Malone TF (1985). Resilience of Ecosystems: Local Surprise and Global Change.

[6] Duffy JE (2007). The functional role of biodiversity in ecosystems: Incorporating trophic complexity. Ecol. Lett..

[7] Lafferty DJR, Belant JL, Phillips DL (2015). Testing the niche variation hypothesis with a measure of body condition. Oikos.

[8] Mangipane LS (2018). Dietary plasticity in a nutrient-rich system does not influence brown bear (*Ursus arctos*) body condition or denning. Polar Biol..

[9] Mangipane LS (2020). Dietary plasticity and the importance of salmon to brown bear (*Ursus arctos*) body size and condition in a low Arctic ecosystem. Polar Biol..

[10] Stumpf RM (2016). Microbiomes, metagenomics, and primate conservation: New strategies, tools, and applications. Biol. Conserv..

[11] McKenney EA, Koelle K, Dunn RR, Yoder AD (2018). The ecosystem services of animal microbiomes. Mol. Ecol..

[12] Kau AL, Ahern PP, Griffin NW, Goodman AL, Gordon JI (2011). Human nutrition, the gut microbiome and the immune system. Nature.

[13] Martin AM, Sun EW, Rogers GB, Keating DJ (2019). The influence of the gut microbiome on host metabolism through the regulation of gut hormone release. Front. Physiol..

[14] Amato KR (2015). The gut microbiota appears to compensate for seasonal diet variation in the wild black howler monkey (*Alouatta pigra*). Microb. Ecol..

[15] Turnbaugh PJ (2006). An obesity-associated gut microbiome with increased capacity for energy harvest. Nature.

[16] Cani PD, Delzenne NM (2009). Interplay between obesity and associated metabolic disorders: New insights into the gut microbiota. Curr. Opin. Pharmacol..

[17] Arinell K (2012). Brown bears (*Ursus arctos*) seem resistant to atherosclerosis­despite highly elevated plasma lipids during hibernation and active state. Clin. Transl. Sci..

[18] Nelson RA (1980). Protein and fat metabolism in hibernating bears. Fed. Proc..

[19] Ley RE (2008). Evolution of mammals and their gut microbes. Science.

[20] McKenney EA, Maslanka M, Rodrigo A, Yoder AD (2018). Bamboo specialists from two mammalian orders (primates, carnivora) share a high number of low-abundance gut microbes. Microb. Ecol..

[21] Edwards MA, Derocher AE, Hobson KA, Branigan M, Nagy JA (2011). Fast carnivores and slow herbivores: Differential foraging strategies among grizzly bears in the Canadian Arctic. Oecologia.

[22] Levi T (2020). Community ecology and conservation of bear-salmon ecosystems. Front. Ecol. Evol..

[23] Milakovic B, Parker KL (2013). Quantifying carnivory by grizzly bears in a multi-ungulate system. J. Wildl. Manage..

[24] Krajmalnik-Brown R, Ilhan Z-E, Kang D-W, DiBaise JK (2012). Effects of gut microbes on nutrient absorption and energy regulation. Nutr. Clin. Pract..

[25] Flint HJ, Scott KP, Duncan SH, Louis P, Forano E (2012). Microbial degradation of complex carbohydrates in the gut. Gut Microbes.

[26] Hashimoto T, Hussien R, Brooks GA (2006). Colocalization of MCT1, CD147, and LDH in mitochondrial inner membrane of L6 muscle cells: Evidence of a mitochondrial lactate oxidation complex. Am. J. Physiol.-Endocrinol. Metab..

[27] Baker S, The HC (2018). Recent insights into Shigella: A major contributor to the global diarrhoeal disease burden. Curr. Opin. Infect. Dis..

[28] Lee K-E (2020). The extracellular vesicle of gut microbial *Paenalcaligenes hominis* is a risk factor for vagus nerve-mediated cognitive impairment. Microbiome.

[29] Waites KB, Schelonka RL, Xiao L, Grigsby PL, Novy MJ (2009). Congenital and opportunistic infections: Ureaplasma species and *Mycoplasma hominis*. Semin. Fetal Neonatal. Med..

[30] Barboza PS, Farley SD, Robbins CT (2011). Whole-body urea cycling and protein turnover during hyperphagia and dormancy in growing bears (*Ursus americanus* and *U. arctos*). Can. J. Zool..

[31] Johanne Hansen M (2015). *Ursidibacter maritimus* gen. nov., sp. nov. and *Ursidibacter arcticus* sp. nov., two new members of the family Pasteurellaceae isolated from the oral cavity of bears. Int. J. Syst. Evol. Microbiol..

[32] Waldram A (2009). Top-down systems biology modeling of host metabotype-microbiome associations in obese rodents. J. Proteome Res..

[33] Hardie JM, Whiley RA, Wood BJB, Holzapfel WH (1995). The genus Streptococcus. The Genera of Lactic Acid Bacteria.

[34] Li F, Wang M, Wang J, Li R, Zhang Y (2019). Alterations to the gut microbiota and their correlation with inflammatory factors in chronic kidney disease. Front. Cell. Infect. Microbiol..

[35] Fox JG, Lee A (1997). The role of Helicobacter species in newly recognized gastrointestinal tract diseases of animals. Lab. Anim. Sci..

[36] McKenney EA, Rodrigo A, Yoder AD (2015). Patterns of gut bacterial colonization in three primate species. PLoS ONE.

[37] Stevens CE, Hume ID (1998). Contributions of microbes in vertebrate gastrointestinal tract to production and conservation of nutrients. Physiol. Rev..

[38] Hilderbrand GV (2018). Plasticity in physiological condition of female brown bears across diverse ecosystems. Polar Biol..

[39] Ley RE (2005). Obesity alters gut microbial ecology. PNAS.

[40] Sommer F (2016). The gut microbiota modulates energy metabolism in the hibernating brown bear ursus arctos. Cell Rep..

[41] Magne F (2020). The firmicutes/bacteroidetes ratio: A relevant marker of gut dysbiosis in obese patients?. Nutrients.

[42] Paine RT (1969). A note on trophic complexity and community stability. Am. Nat..

[43] Amato KR (2019). Evolutionary trends in host physiology outweigh dietary niche in structuring primate gut microbiomes. ISME J..

[44] Trujillo SM (2022). Intrinsic and extrinsic factors influence on an omnivore’s gut microbiome. PLoS ONE.

[45] Hilderbrand GV (2018). Body size and lean mass of brown bears across and within four diverse ecosystems. J. Zool..

[46] Wilson RR, Gustine DD, Joly K (2014). Evaluating potential effects of an industrial road on winter habitat of caribou in North-Central Alaska. Arctic.

[47] Gasaway WC (1992). The role of predation in limiting moose at low densities in Alaska and Yukon and implications for conservation. Wildl. Monogr..

[48] Taylor WP, Reynolds HV, Ballard WB (1989). Immobilization of grizzly bears with tiletamine hydrochloride and zolazepam hydrochloride. J. Wildl. Manage..

[49] Farley SD, Robbins CT (1994). Development of two methods to estimate body composition of bears. Can. J. Zool..

[50] Hilderbrand GV, Robbins CT, Farley SD (1998). Response: Use of stable isotopes to determine diets of living and extinct bears. Can. J. Zool..

[51] McKenney EA, Greene LK, Drea CM, Yoder AD (2017). Down for the count: Cryptosporidium infection depletes the gut microbiome in Coquerel’s sifakas. Microb. Ecol. Health Dis..

[52] Callahan BJ (2016). DADA2: High-resolution sample inference from Illumina amplicon data. Nat. Methods.

[53] Quast C (2013). The SILVA ribosomal RNA gene database project: Improved data processing and web-based tools. Nucleic Acids Res..

[54] Willis AD (2019). Rarefaction, alpha diversity, and statistics. Front. Microbiol..

[55] Beule L, Karlovsky P (2020). Improved normalization of species count data in ecology by scaling with ranked subsampling (SRS): Application to microbial communities. PeerJ.

[56] Galand PE, Casamayor EO, Kirchman DL, Lovejoy C (2009). Ecology of the rare microbial biosphere of the Arctic Ocean. Proc. Natl. Acad. Sci. U.S.A..

[57] Liu L, Yang J, Yu Z, Wilkinson DM (2015). The biogeography of abundant and rare bacterioplankton in the lakes and reservoirs of China. ISME J..

[58] Segata N (2011). Metagenomic biomarker discovery and explanation. Genome Biol..

[59] Hill MO (1973). Diversity and evenness: A unifying notation and its consequences. Ecology.

[60] Faith DP (1992). Conservation evaluation and phylogenetic diversity. Biol. Conserv..

[61] Simpson EH (1949). Measurement of diversity. Nature.

[62] Lozupone C, Knight R (2006). UniFrac: A new phylogenetic method for comparing microbial communities. Appl. Environ. Microbiol..

[63] Hamidi B, Wallace K, Vasu C, Alekseyenko AV (2019). Wd∗$W_d_*-test: Robust distance-based multivariate analysis of variance. Microbiome.

[64] Alekseyenko AV (2016). Multivariate Welch t-test on distances. Bioinformatics.

