# Supplementary material for: Correlating gut microbial membership to brown bear health metrics
Source: Sci Rep. 2022 Sep 22;12:15415. doi: 10.1038/s41598-022-19527-4 (PMC9499961; doi:10.1038/s41598-022-19527-4)
Supplement: Supplementary file 1 — Supplementary Information. [file 41598_2022_19527_MOESM1_ESM.pdf]

Correlating gut microbial membership to brown bear health metrics

Sarah M. Trujillo[a*], Erin A. McKenney[b], Grant V. Hilderbrand[c], Lindsey S. Mangipane[d], Matthew C. Rogers[e], Kyle Joly[f], David D. Gustine[d], Joy A. Erlenbach[g], Buck A. Mangipane[h], Diana J. R. Lafferty[a]

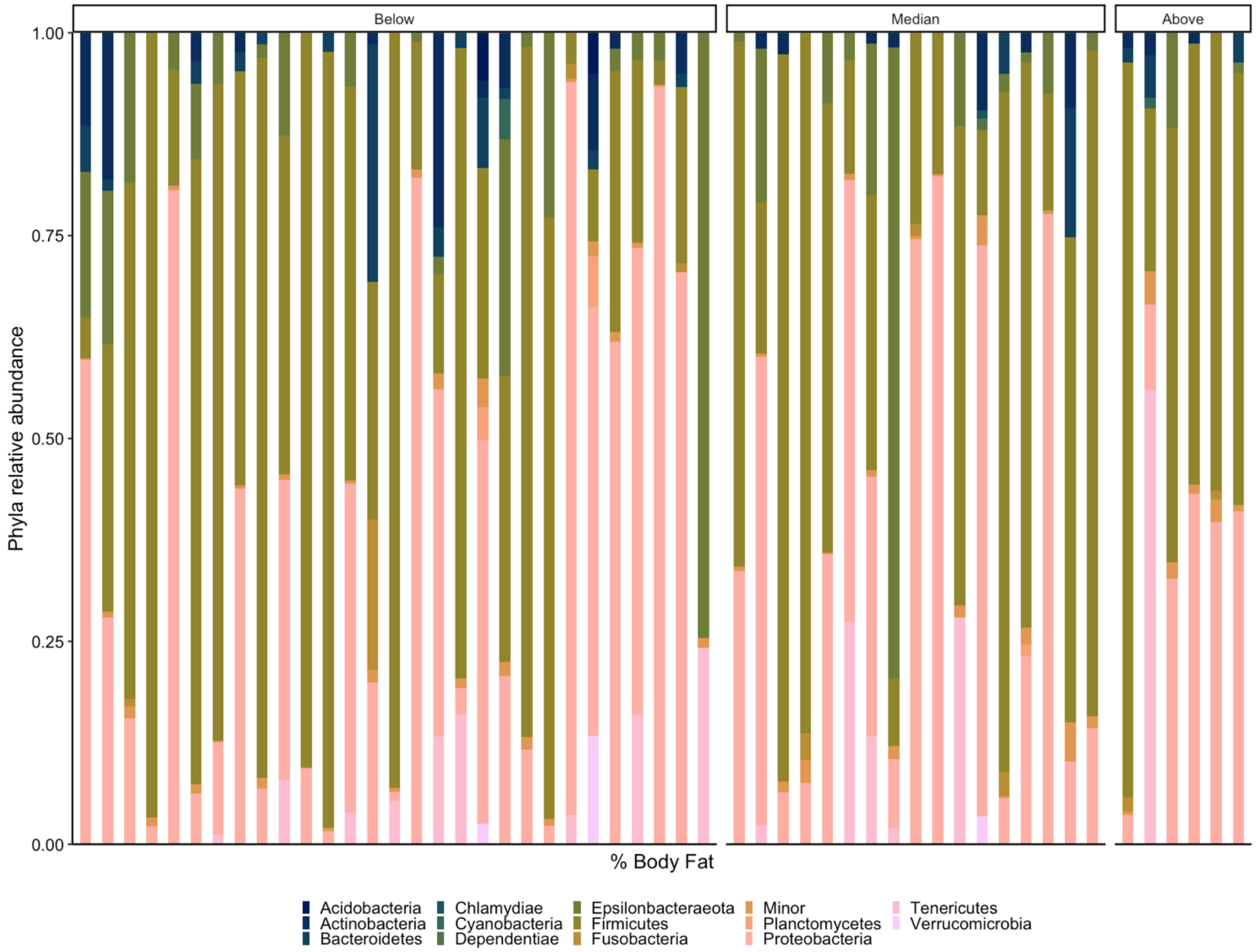

Supplementary Figure S1. Relative abundance of the major bacterial phyla found in Alaskan brown bears (*Ursus arctos*) across percent body fat categories: below median, median, and above median. We include all major taxa occurring at ≥1% relative abundance; "minor" taxa are those occurring at <1% relative abundance. Categorization of body metrics created using median splitting.

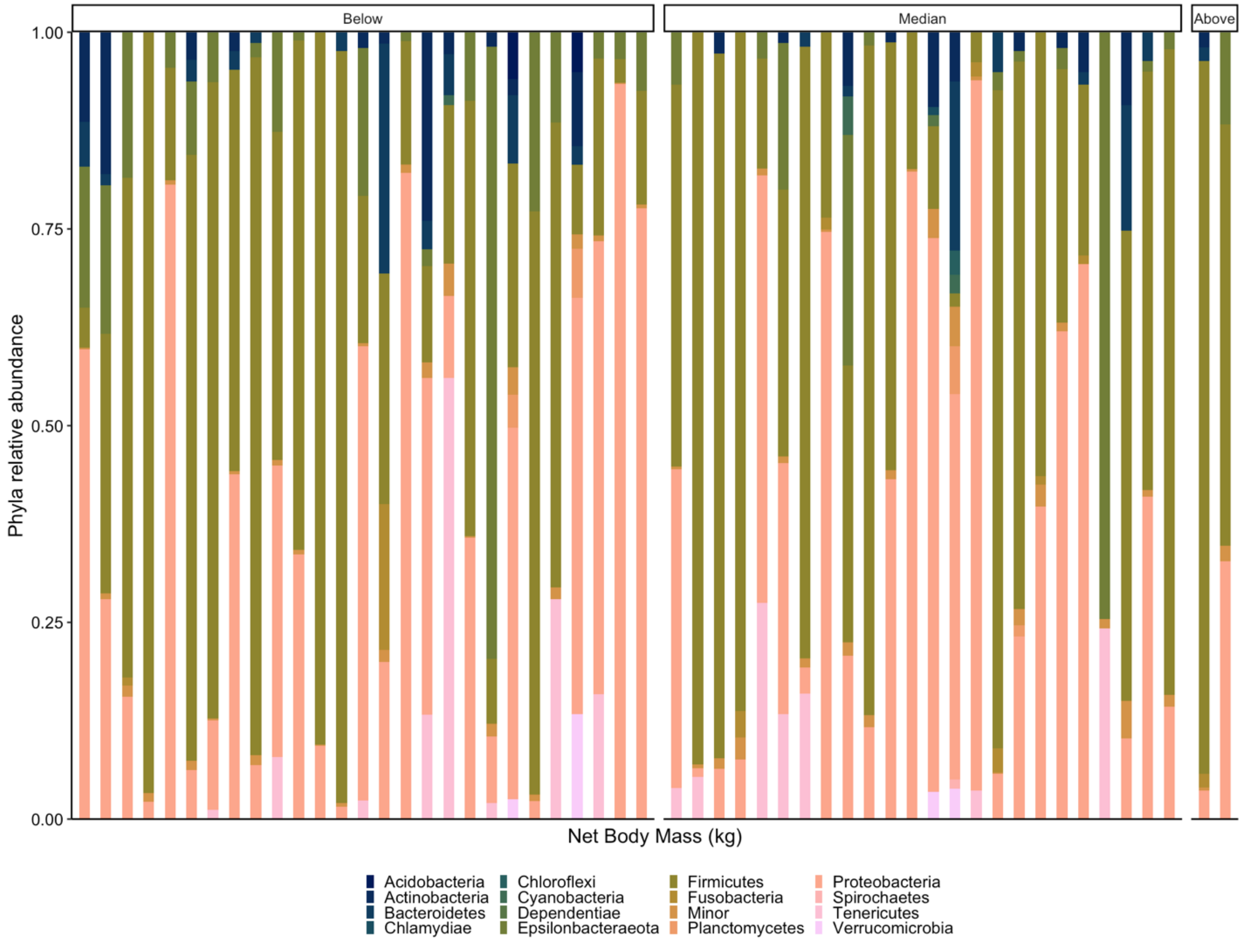

Supplementary Figure S2. Relative abundance of the major bacterial phyla found in Alaskan brown bears (*Ursus arctos*) across percent net body mass categories: below median, median, and above median. We include all major taxa occurring at ≥1% relative abundance; "minor" taxa are those occurring at <1% relative abundance. Categorization of body metrics created using median splitting.

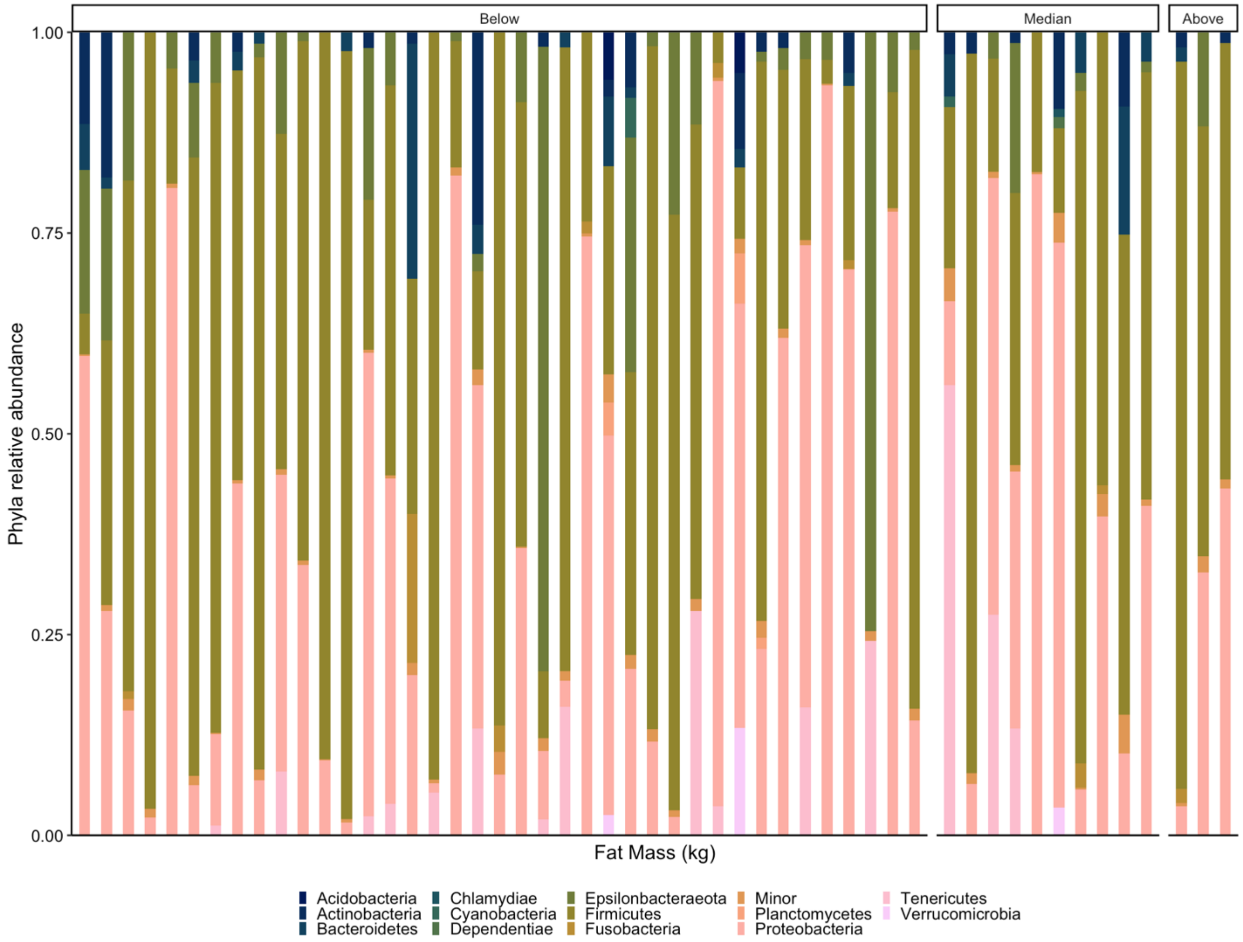

Supplementary Figure S3. Relative abundance of the major bacterial phyla found in Alaskan brown bears (*Ursus arctos*) across fat mass categories: below median, median, and above median. We include all major taxa occurring at ≥1% relative abundance; "minor" taxa are those occurring at <1% relative abundance. Categorization of body metrics created using median splitting.

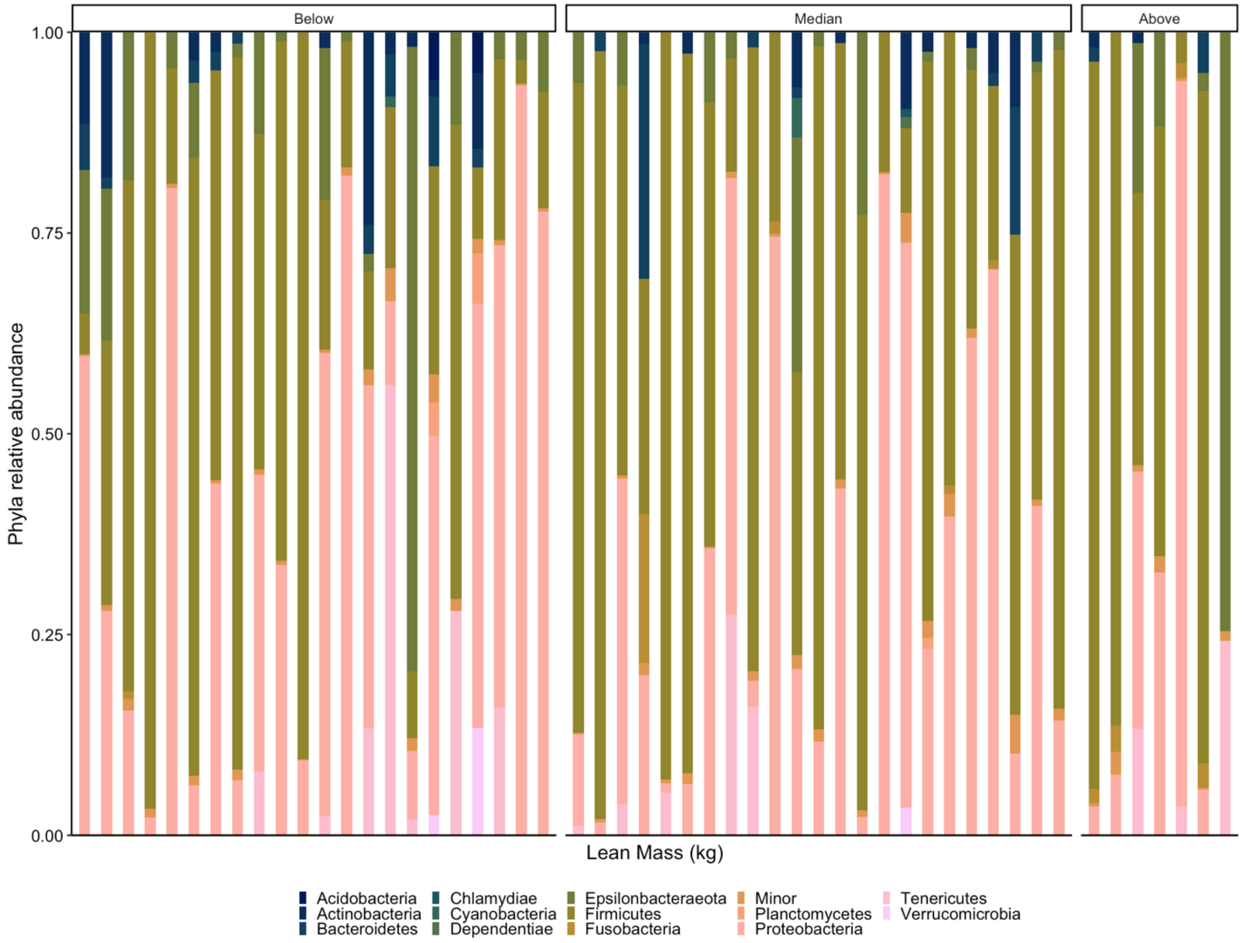

Supplementary Figure S4. Relative abundance of the major bacterial phyla found in Alaskan brown bears (*Ursus arctos*) across lean mass categories: below median, median, and above median. We include all major taxa occurring at ≥1% relative abundance; "minor" taxa are those occurring at <1% relative abundance. Categorization of body metrics created using median splitting.

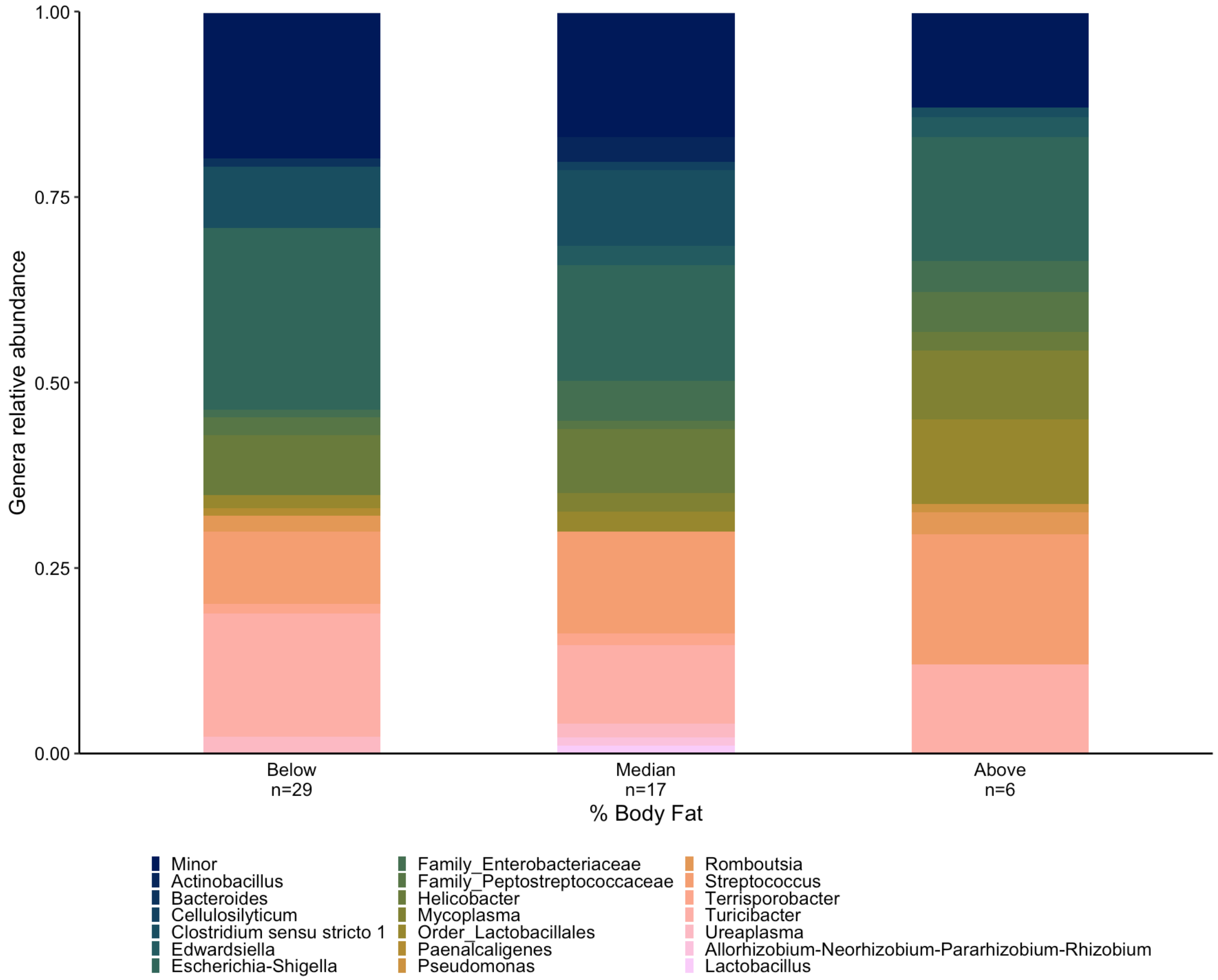

Supplementary Figure S5. Relative abundance of the major bacterial genera found across percent body fat categories in Alaskan brown bear (*Ursus arctos*). We include all major taxa occurring at ≥1% relative abundance; “minor” taxa are those occurring at <1% relative abundance. Categorization of body metrics created using median splitting.

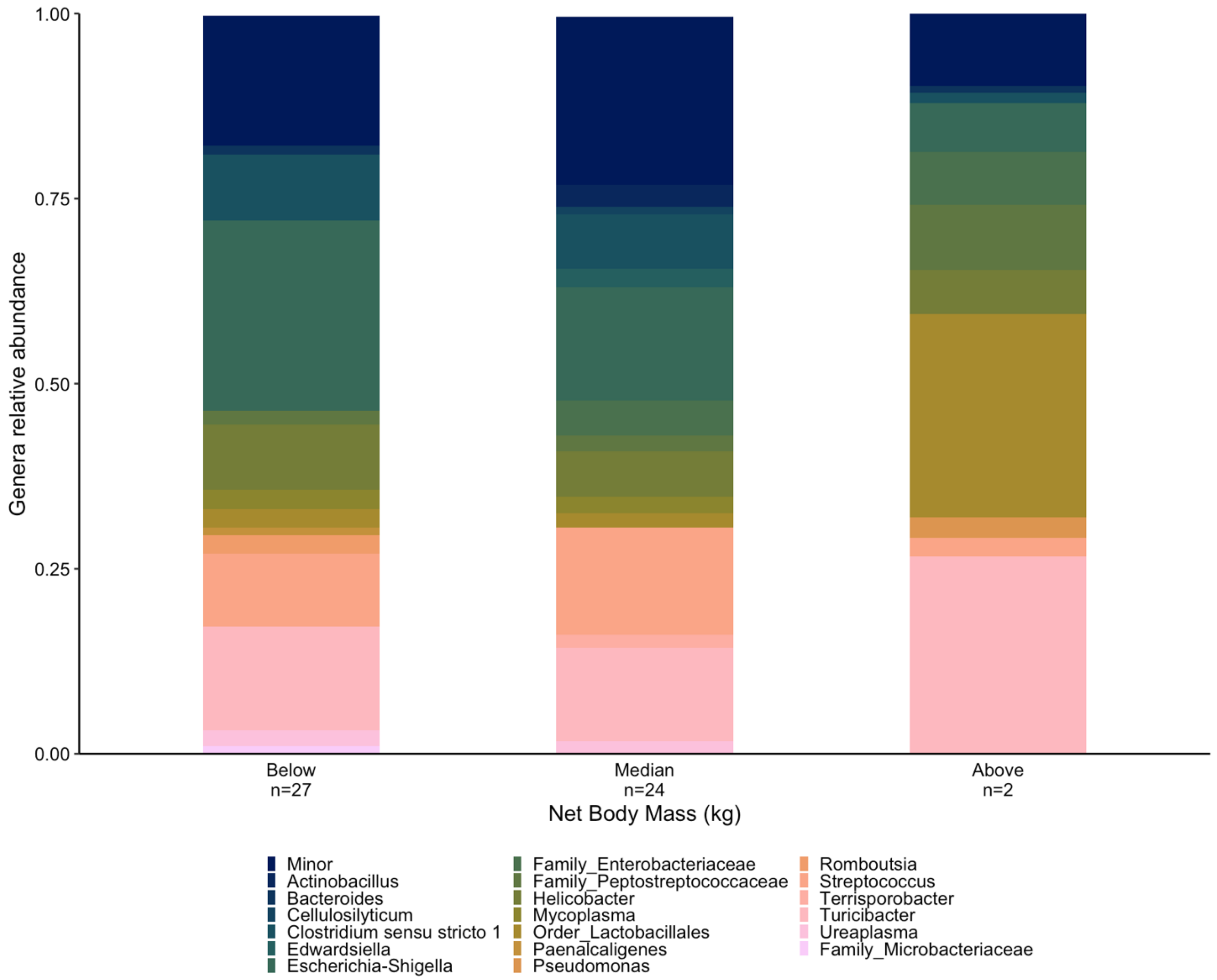

Supplementary Figure S6. Relative abundance of the major bacterial genera found across net body mass categories in Alaskan brown bear (*Ursus arctos*). We include all major taxa occurring at ≥1% relative abundance; “minor” taxa are those occurring at <1% relative abundance. Categorization of body metrics created using median splitting.

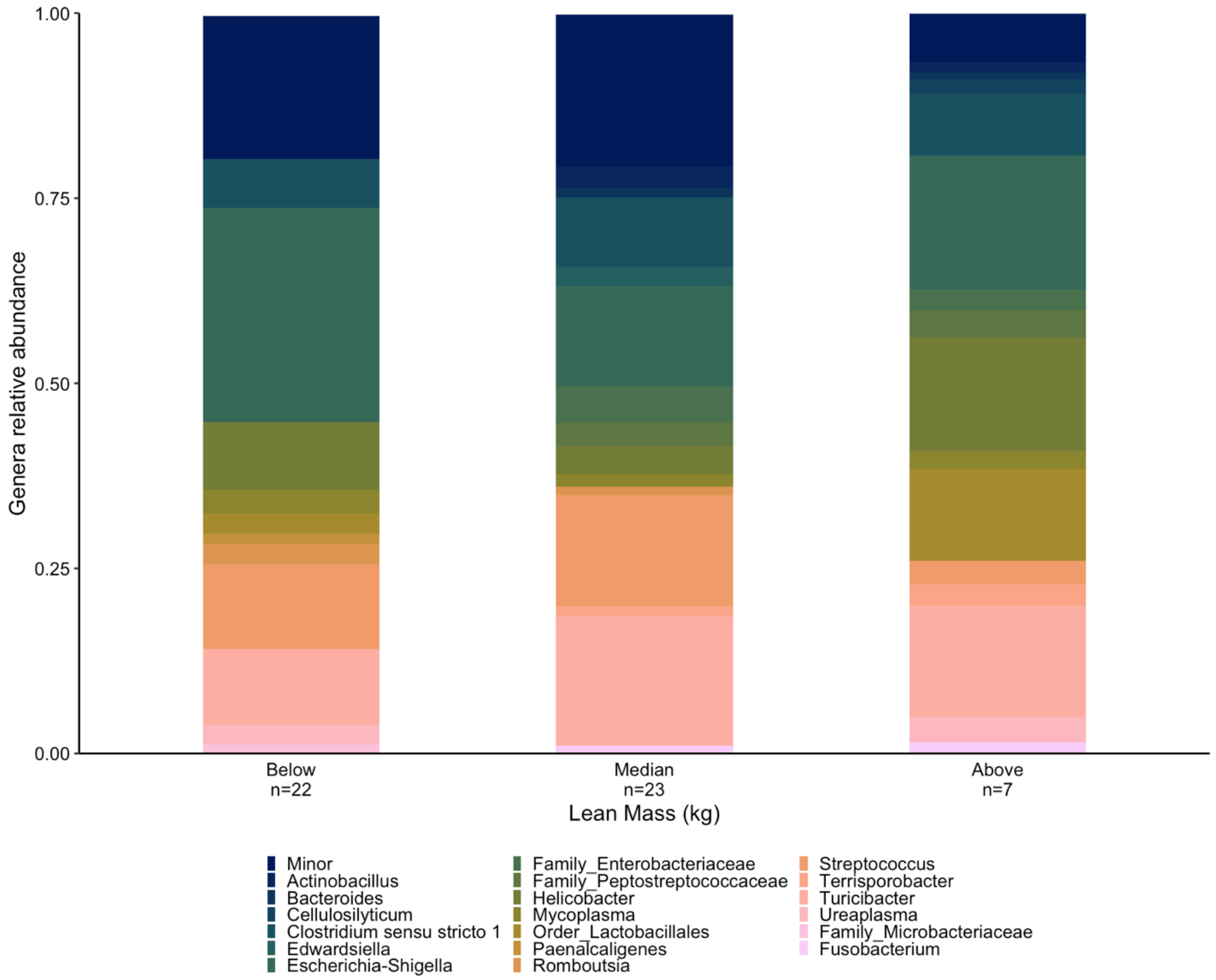

Supplementary Figure S7. Relative abundance of the major bacterial genera found across lean mass categories in Alaskan brown bear (*Ursus arctos*). We include all major taxa occurring at ≥1% relative abundance; "minor" taxa are those occurring at <1% relative abundance. Categorization of body metrics created using median splitting.

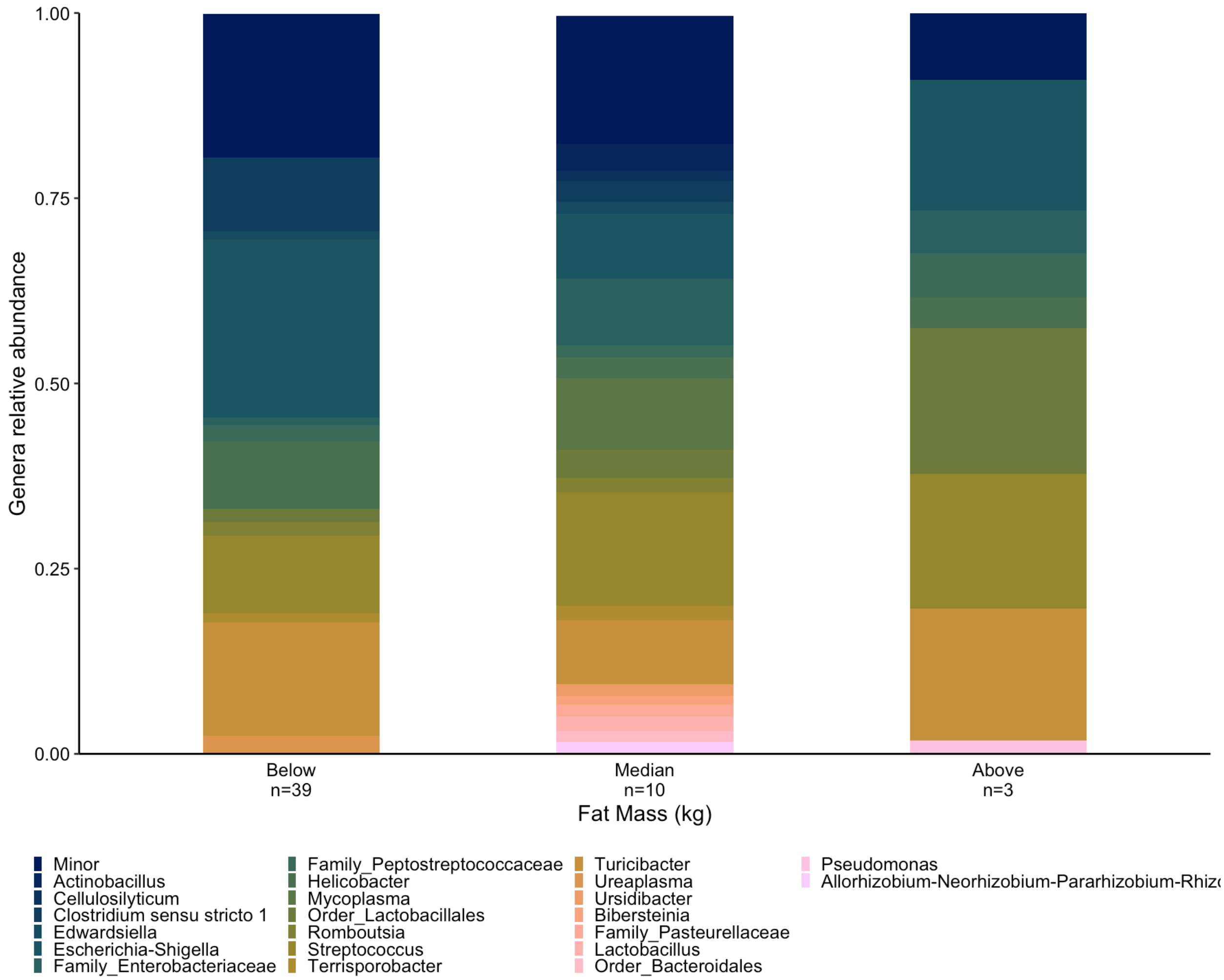

Supplementary Figure S8. Relative abundance of the major bacterial genera found across fat mass categories in Alaskan brown bear (*Ursus arctos*). We include all major taxa occurring at ≥1% relative abundance; “minor” taxa are those occurring at <1% relative abundance. Categorization of body metrics created using median splitting.

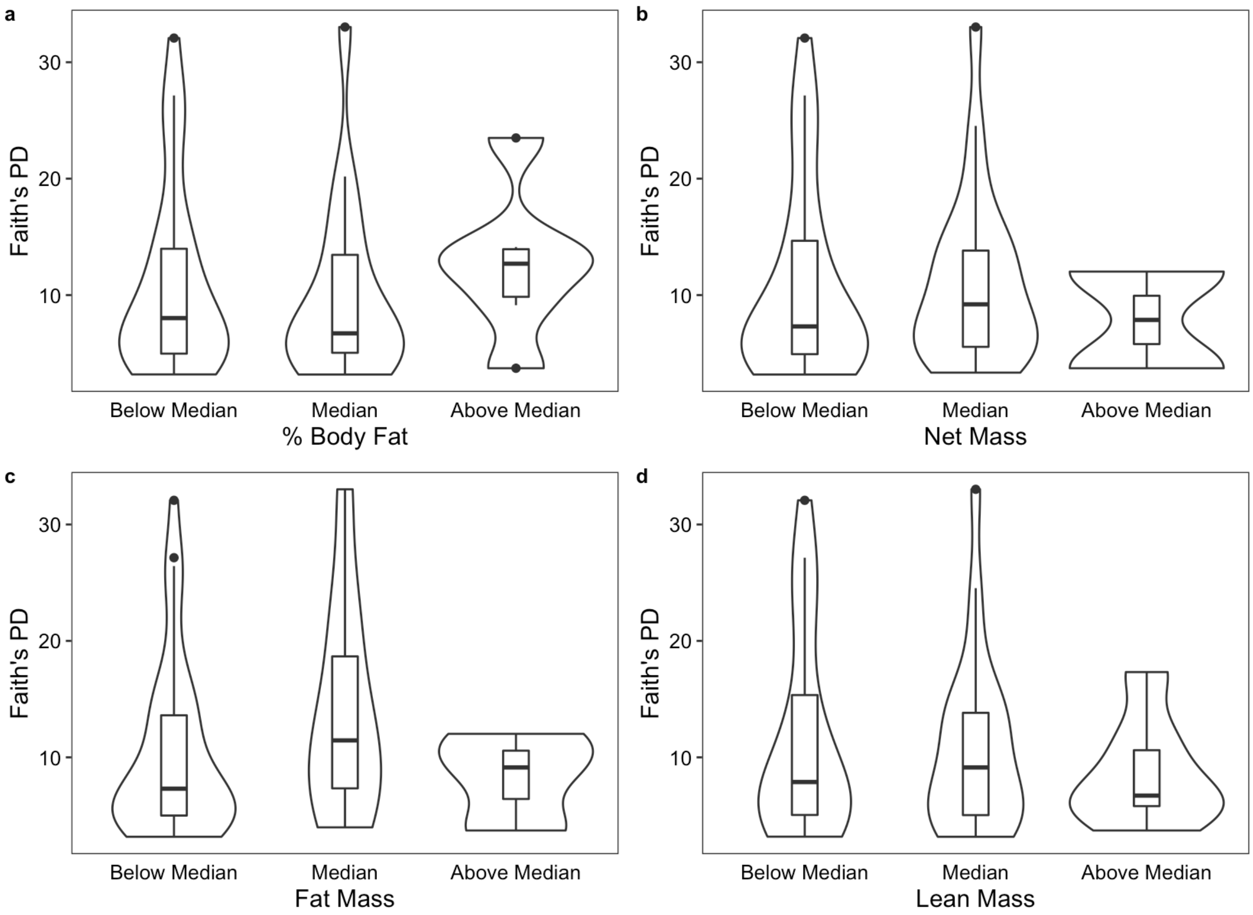

Supplementary Figure S9. Violin plots summarizing Faith’s phylogenetic diversity of brown bear (*Ursus arctos*) gut microbiomes for each body condition category: **a)** percent body fat; **b)** net body mass; **c)** fat mass; and **d)** lean mass.

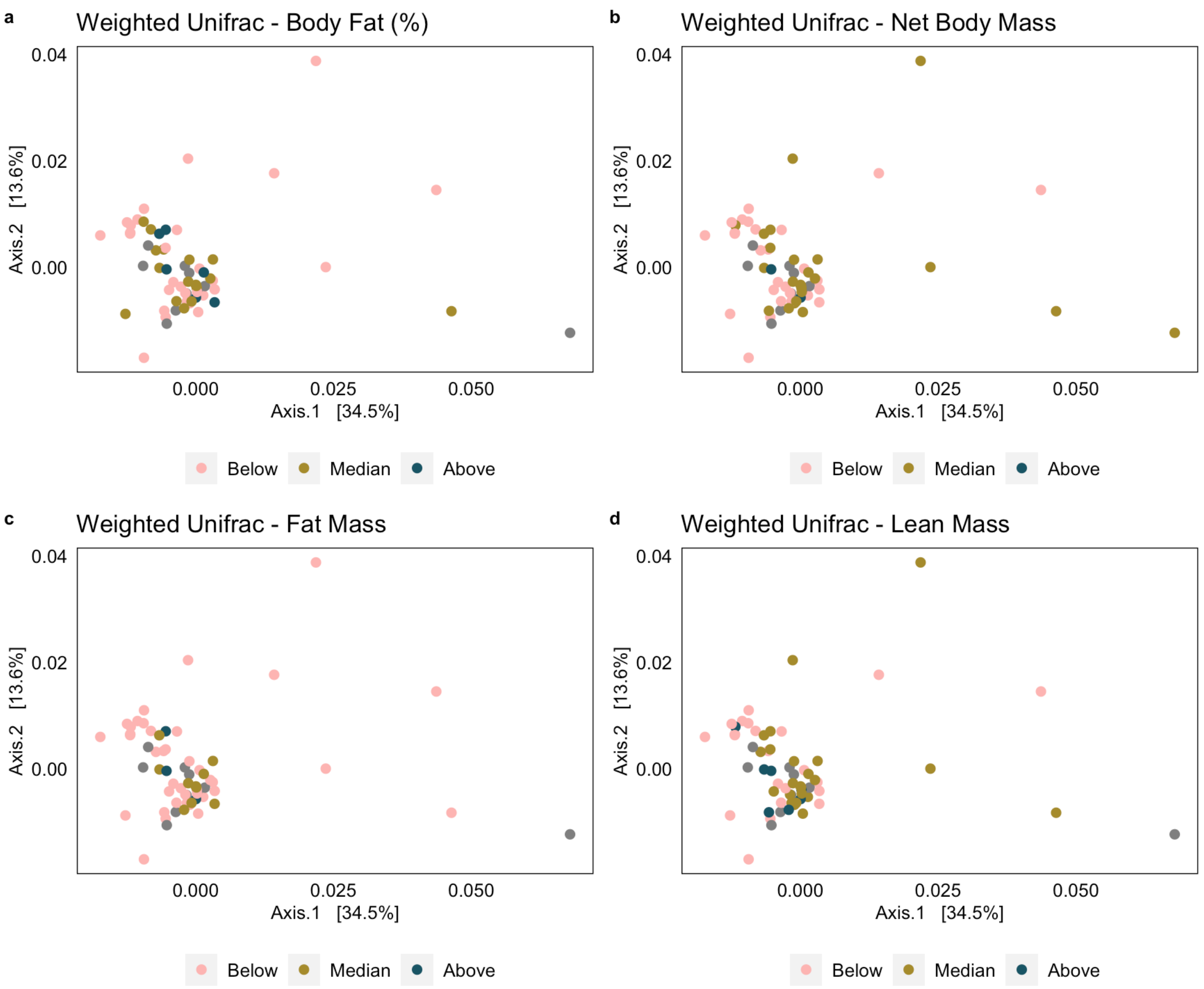

Supplementary Figure S10. Gut microbial beta diversity among Alaskan brown bears (*Ursus arctos*). Principle Coordinate Analysis plots of weighted UniFrac distances in brown bears with varying **a)** percent body fat, **b)** net body mass, **c)** fat mass, and **d)** lean mass.

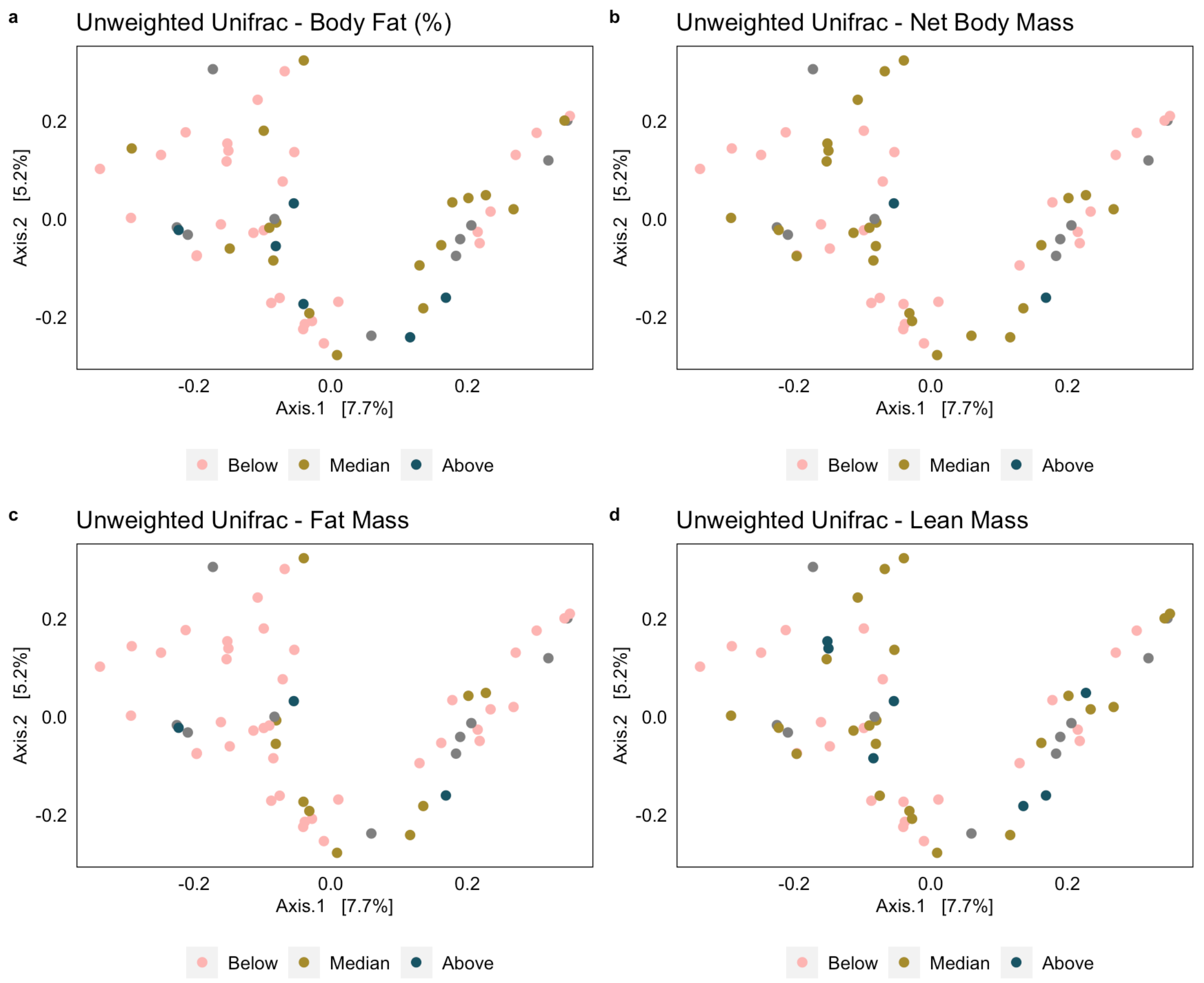

Supplementary Figure S11. Gut microbial beta diversity among Alaskan brown bears (*Ursus arctos*). Principle Coordinate Analysis plots of weighted UniFrac distances in brown bears with varying **a)** percent body fat, **b)** net body mass, **c)** fat mass, and **d)** lean mass.

Supplementary Table S1. Gut microbial community composition in Alaskan brown bear (*Ursus arctos*). Total relative abundance of major genera (≥1%) within bears sampled.

Phylum	Genus	Abundance	sd	Total
Actinobacteria	Minor (total)	2.277%	NA	2.277%
Bacteroidetes	Minor (total)	2.098%	NA	2.098%
Epsilonbacteraeota	*Helicobacter*	7.002%	14.027%	7.263%
	Minor (total)	0.261%	NA	
Firmicutes	*Clostridium sensu stricto 1*	8.901%	14.793%	49.458%
	Family Peptostreptococcaceae	2.304%	4.843%	
	Order Lactobacillales	2.773%	6.825%	
	Romboutsia	1.816%	5.702%	
	Streptococcus	11.120%	18.692%	
	Terrisporobacter	1.255%	2.540%	
	Turicibacter	17.189%	24.334%	

	Minor (total)	4.100%	NA	
Proteobacteria	*Actinobacillus*	1.233%	4.659%	31.181%
	Escherichia-Shigella	19.085%	24.621%	
	Family Enterobacteriaceae	2.427%	9.847%	
	Minor (total)	8.436%	NA	
Tenericutes	*Mycoplasma*	2.378%	8.507%	5.471%
	Ureaplasma	3.071%	9.998%	
	Minor (total)	0.022%	NA	
Minor	Minor (total)	2.252%	NA	2.252%

Supplementary Table S2. Mean abundance of the six major bacterial phyla (≥1% relative abundance) in brown bear (*Ursus arctos*) gut microbiomes among different body condition categories.

a. Actinobacteria

**Body Condition**	**N**	**Abundance**	**sd**	**se**	**ci**
Below Median Body Fat	29	0.031	0.058	0.011	0.022
Median Body Fat	17	0.018	0.030	0.007	0.015
Above Median Body Fat	6	0.013	0.009	0.004	0.010
Below Median Net Mass	27	0.030	0.059	0.011	0.023
Median Net Mass	24	0.021	0.030	0.006	0.013
Above Median Net Mass	2	0.014	0.007	0.005	0.065
Below Median Lean Mass	22	0.037	0.064	0.014	0.028
Median Lean Mass	23	0.019	0.029	0.006	0.013
Above Median Lean Mass	7	0.008	0.007	0.003	0.006
Below Median Fat Mass	39	0.025	0.051	0.008	0.017
Median Fat Mass	10	0.027	0.037	0.012	0.026

b. Bacteroidetes

**Body Condition**	**N**	**Abundance**	**sd**	**se**	**ci**
Below Median Body Fat	29	0.023	0.055	0.010	0.021
Median Body Fat	17	0.015	0.039	0.009	0.020
Above Median Body Fat	6	0.020	0.021	0.008	0.022
Below Median Net Mass	27	0.025	0.058	0.011	0.023
Median Net Mass	24	0.024	0.053	0.011	0.022
Above Median Net Mass	2	0.013	0.006	0.004	0.051
Below Median Lean Mass	22	0.017	0.023	0.005	0.010
Median Lean Mass	23	0.026	0.067	0.014	0.029
Above Median Lean Mass	7	0.013	0.018	0.007	0.016
Below Median Fat Mass	39	0.018	0.048	0.008	0.016
Median Fat Mass	10	0.032	0.049	0.016	0.035

c. Epsilonbacteraeota

**Body Condition**	**N**	**Abundance**	**sd**	**se**	**ci**
Below Median Body Fat	29	0.083	0.151	0.028	0.057
Median Body Fat	17	0.091	0.188	0.046	0.100

Above Median Body Fat	6	0.026	0.045	0.018	0.047
Below Median Net Mass	27	0.093	0.154	0.030	0.061
Median Net Mass	24	0.061	0.161	0.033	0.068
Above Median Net Mass	2	0.059	0.082	0.058	0.737
Below Median Lean Mass	22	0.097	0.167	0.036	0.074
Median Lean Mass	23	0.019	0.029	0.006	0.013
Above Median Lean Mass	7	0.154	0.271	0.102	0.251
Below Median Fat Mass	39	0.095	0.174	0.028	0.056
Median Fat Mass	10	0.028	0.057	0.018	0.040
Above Median Fat Mass	3	0.042	0.065	0.038	0.162

d. Firmicutes

Body Condition	N	Abundance	sd	se	ci
Below Median Body Fat	29	0.458	0.332	0.062	0.127
Median Body Fat	17	0.465	0.301	0.073	0.155
Above Median Body Fat	6	0.547	0.223	0.091	0.234
Below Median Net Mass	27	0.434	0.315	0.061	0.125
Median Net Mass	24	0.473	0.312	0.064	0.132
Above Median Net Mass	2	0.721	0.262	0.185	2.353
Below Median Lean Mass	22	0.380	0.306	0.065	0.136
Median Lean Mass	23	0.548	0.274	0.057	0.118
Above Median Lean Mass	7	0.503	0.386	0.146	0.357
Below Median Fat Mass	39	0.464	0.319	0.051	0.103
Median Fat Mass	10	0.439	0.289	0.092	0.207
Above Median Fat Mass	3	0.662	0.212	0.122	0.526

e. Proteobacteria

Body Condition	N	Abundance	sd	se	ci
Below Median Body Fat	29	0.345	0.300	0.056	0.114
Median Body Fat	17	0.350	0.288	0.070	0.148
Above Median Body Fat	6	0.285	0.171	0.070	0.179
Below Median Net Mass	27	0.342	0.284	0.055	0.112
Median Net Mass	24	0.356	0.284	0.058	0.120
Above Median Net Mass	2	0.182	0.206	0.146	1.850
Below Median Lean Mass	22	0.388	0.290	0.062	0.129
Median Lean Mass	23	0.322	0.263	0.055	0.114
Above Median Lean Mass	7	0.246	0.319	0.121	0.295
Below Median Fat Mass	39	0.342	0.292	0.047	0.095
Median Fat Mass	10	0.352	0.275	0.087	0.197
Above Median Fat Mass	3	0.265	0.205	0.118	0.509

f. Tenericutes

Body Condition	N	Abundance	sd	se	ci
Below Median Body Fat	29	0.032	0.063	0.012	0.024
Median Body Fat	17	0.043	0.094	0.023	0.048
Above Median Body Fat	6	0.094	0.229	0.093	0.240
Below Median Net Mass	27	0.047	0.121	0.023	0.048
Median Net Mass	24	0.040	0.079	0.016	0.034

Below Median Lean Mass	22	0.057	0.133	0.028	0.059
Median Lean Mass	23	0.024	0.065	0.014	0.029
Above Median Lean Mass	7	0.059	0.094	0.036	0.087
Below Median Fat Mass	39	0.032	0.069	0.011	0.022
Median Fat Mass	10	0.098	0.186	0.059	0.133

Supplementary Table S3. One-way analysis of variance test for significant differences in brown bear (*Ursus arctos*) gut microbiome major phyla abundance (≥1% relative abundance) between body metrics. No significant differences.

a. Actinobacteria

	df	Sum Sq	Mean Sq	F value	Pr(>F)
Body Fat	2	0.002	0.001	0.437	0.649
Residuals	46	0.107	0.002		
Net Mass	2	0.001	0.001	0.182	0.834
Residuals	47	0.110	0.002		
Lean Mass	2	0.005	0.003	1.134	0.331
Residuals	46	0.104	0.002		
Fat Mass	2	0.001	0.001	0.124	0.884
Residuals	46	0.109	0.002		

b. Bacteroidetes

	df	Sum Sq	Mean Sq	F value	Pr(>F)
Body Fat	2	0.001	0.001	0.244	0.784
Residuals	46	0.110	0.002		
Net Mass	2	0.001	0.001	0.062	0.94
Residuals	47	0.148	0.003		
Lean Mass	2	0.002	0.001	0.327	0.723
Residuals	46	0.110	0.002		
Fat Mass	2	0.002	0.001	0.370	0.693
Residuals	46	0.110	0.002		

c. Epsilonbacteraeota

	df	Sum Sq	Mean Sq	F value	Pr(>F)
Body Fat	2	0.024	0.012	0.457	0.636
Residuals	46	1.186	0.026		
Net Mass	2	0.007	0.004	0.144	0.866
Residuals	47	1.201	0.026		
Lean Mass	2	0.068	0.034	1.364	0.266
Residuals	46	1.141	0.025		
Fat Mass	2	0.040	0.020	0.778	0.465
Residuals	46	1.170	0.025		

d. Tenericutes					
	**df**	**Sum Sq**	**Mean Sq**	**F value**	**Pr(>F)**
Body Fat	2	0.078	0.039	2.412	0.114
Residuals	21	0.341	0.016		
Net Mass	2	0.024	0.024	1.377	0.253
Residuals	23	0.403	0.018		
Lean Mass	2	0.052	0.026	1.491	0.248
Residuals	21	0.367	0.017		
Fat Mass	2	0.040	0.040	2.315	0.142
Residuals	22	0.380	0.017		

Supplementary Table S4. Kruskal-Wallis rank sum test for significant differences in brown bear (*Ursus arctos*) gut microbiome major phyla abundance (≥1% relative abundance) between body metrics for non-parametric data. P-value adjusted with Bonferroni correction. No significant differences.

a. Firmicutes			
	**K-W chi-squared**	**df**	**P value**
Body Fat	0.489	2	0.783
Net Mass	1.621	2	0.445
Lean Mass	3.414	2	0.181
Fat Mass	1.288	2	0.525
**b. Proteobacteria**			
	**K-W chi-squared**	**df**	**P value**
Body Fat	0.065	2	0.968
Net Mass	0.814	2	0.666
Lean Mass	2.316	2	0.314
Fat Mass	0.184	2	0.912

Supplementary Table S5. Mean abundance of the five dominant bacterial phyla abundance (≥10% mean abundance) in brown bear (*Ursus arctos*) gut microbiomes among different body condition categories.

a. ***Escherichia-Shigella***					
**Body Condition**	**N**	**Abundance**	**sd**	**se**	**ci**
Below Median Body Fat	29	0.245	0.295	0.055	0.112
Median Body Fat	17	0.155	0.222	0.054	0.114
Above Median Body Fat	6	0.167	0.180	0.074	0.189
Below Median Net Mass	27	0.257	0.289	0.056	0.114
Median Net Mass	24	0.153	0.227	0.046	0.098
Above Median Net Mass	2	0.066	0.061	0.043	0.550
Below Median Lean Mass	22	0.289	0.306	0.065	0.136
Median Lean Mass	23	0.135	0.170	0.035	0.073
Above Median Lean Mass	7	0.181	0.322	0.122	0.298
Below Median Fat Mass	39	0.240	0.285	0.046	0.092

Median Fat Mass	10	0.087	0.125	0.040	0.090
Above Median Fat Mass	3	0.176	0.196	0.113	0.487

b. ***Streptococcus***

**Body Condition**	**N**	**Abundance**	**sd**	**se**	**ci**
Below Median Body Fat	29	0.097	0.159	0.029	0.060
Median Body Fat	17	0.137	0.247	0.060	0.127
Above Median Body Fat	6	0.175	0.236	0.096	0.248
Below Median Net Mass	27	0.099	0.168	0.032	0.066
Median Net Mass	24	0.145	0.232	0.047	0.098
Above Median Net Mass	2	0.026	0.022	0.016	0.197
Below Median Lean Mass	22	0.114	0.181	0.039	0.080
Median Lean Mass	23	0.151	0.236	0.049	0.102
Above Median Lean Mass	7	0.031	0.031	0.012	0.029
Below Median Fat Mass	39	0.106	0.186	0.030	0.060
Median Fat Mass	10	0.153	0.238	0.075	0.170
Above Median Fat Mass	3	0.182	0.272	0.157	0.675

c. ***Turicibacter***

**Body Condition**	**N**	**Abundance**	**sd**	**se**	**ci**
Below Median Body Fat	29	0.166	0.237	0.044	0.090
Median Body Fat	17	0.106	0.151	0.037	0.078
Above Median Body Fat	6	0.120	0.210	0.086	0.221
Below Median Net Mass	27	0.141	0.216	0.042	0.085
Median Net Mass	24	0.126	0.190	0.039	0.080
Above Median Net Mass	2	0.266	0.375	0.265	3.367
Below Median Lean Mass	22	0.104	0.168	0.036	0.075
Median Lean Mass	23	0.175	0.240	0.050	0.104
Above Median Lean Mass	7	0.150	0.216	0.082	0.200
Below Median Fat Mass	39	0.153	0.220	0.035	0.071
Median Fat Mass	10	0.864	0.121	0.038	0.086
Above Median Fat Mass	3	0.178	0.306	0.177	0.760

d. ***Clostridium sensu stricto 1***

**Body Condition**	**N**	**Abundance**	**sd**	**se**	**ci**
Below Median Body Fat	29	0.082	0.134	0.025	0.051
Median Body Fat	17	0.102	0.174	0.042	0.089
Above Median Body Fat	6	0.013	0.016	0.006	0.017
Below Median Net Mass	27	0.089	0.151	0.029	0.060
Median Net Mass	24	0.073	0.135	0.028	0.057
Above Median Net Mass	2	0.014	0.020	0.014	0.176
Below Median Lean Mass	22	0.066	0.137	0.029	0.061
Median Lean Mass	23	0.094	0.146	0.030	0.063
Above Median Lean Mass	7	0.084	0.155	0.059	0.144
Below Median Fat Mass	39	0.099	0.158	0.025	0.051
Median Fat Mass	10	0.028	0.040	0.013	0.028

d. ***Helicobacter***

**Body Condition**	**N**	**Abundance**	**sd**	**se**	**ci**

Below Median Body Fat	29	0.081	0.151	0.028	0.057
Median Body Fat	17	0.086	0.175	0.042	0.090
Above Median Body Fat	6	0.025	0.045	0.018	0.047
Below Median Net Mass	27	0.089	0.145	0.028	0.057
Median Net Mass	24	0.060	0.161	0.033	0.068
Above Median Net Mass	2	0.059	0.082	0.058	0.737
Below Median Lean Mass	22	0.091	0.156	0.033	0.069
Median Lean Mass	23	0.038	0.075	0.016	0.032
Above Median Lean Mass	7	0.153	0.271	0.102	0.251
Below Median Fat Mass	39	0.091	0.169	0.027	0.055
Median Fat Mass	10	0.028	0.057	0.018	0.040
Above Median Fat Mass	3	0.042	0.065	0.038	0.163

Supplementary Table S6. One-way analysis of variance test for significant differences in brown bear (*Ursus arctos*) gut microbiome dominant genera abundance (≥10% mean abundance) between body metrics. No significant differences.

a. ***Streptococcus***

	**df**	**Sum Sq**	**Mean Sq**	**F value**	**Pr(>F)**
Body Fat	2	0.002	0.001	0.437	0.649
Residuals	46	0.107	0.002		
Net Mass	2	0.001	0.001	0.182	0.834
Residuals	47	0.110	0.002		
Lean Mass	2	0.005	0.003	1.134	0.331
Residuals	46	0.104	0.002		
Fat Mass	2	0.001	0.001	0.124	0.884
Residuals	46	0.109	0.002		

Supplementary Table S7. Kruskal-Wallis rank sum test for significant differences in brown bear (*Ursus arctos*) gut microbiome dominant genera (≥10% mean abundance) between body metrics for non-parametric data. P-value adjusted with Bonferroni correction. No significant differences.

a. ***Escherichia-Shigella***

	**K-W chi-squared**	**df**	**P value**
Body Fat	0.731	2	0.694
Net Mass	1.997	2	0.368
Lean Mass	2.084	2	0.353
Fat Mass	1.779	2	0.411

b. ***Turicibacter***

	**K-W chi-squared**	**df**	**P value**
Body Fat	0.678	2	0.712
Net Mass	0.610	2	0.737
Lean Mass	0.428	2	0.808
Fat Mass	0.392	2	0.822

c. *Clostridium sensu stricto 1*			
	**K-W chi-squared**	**df**	**P value**
Body Fat	1.361	2	0.506
Net Mass	0.196	2	0.907
Lean Mass	0.126	2	0.939
Fat Mass	1.382	2	0.501

d. *Helicobacter*			
	**K-W chi-squared**	**df**	**P value**
Body Fat	1.123	2	0.571
Net Mass	3.649	2	0.161
Lean Mass	2.863	2	0.239
Fat Mass	1.335	2	0.513

Supplementary Table S8. Alpha diversity values of brown bear (*Ursus arctos*) gut microbiomes.

a. Faith's PD					
**Body Condition**	**N**	**Diversity**	**sd**	**se**	**ci**
Below Median Body Fat	29	11.022	8.048	1.494	3.061
Median Body Fat	17	9.906	7.752	1.880	3.985
Above Median Body Fat	6	12.644	6.526	2.664	6.849
Below Median Net Mass	27	10.871	8.300	1.597	3.283
Median Net Mass	23	11.049	7.300	1.522	3.157
Above Median Net Mass	2	7.867	5.869	4.150	52.729
Below Median Lean Mass	22	11.649	8.776	1.871	3.891
Median Lean Mass	23	10.737	7.456	1.555	3.224
Above Median Lean Mass	7	8.593	4.589	1.734	4.244
Below Median Fat Mass	39	10.262	7.419	1.188	2.405
Median Fat Mass	10	13.843	9.228	2.918	6.601
Above Median Fat Mass	3	8.248	4.202	2.426	10.439

b. Shannon diversity					
**Body Condition**	**N**	**Diversity**	**sd**	**se**	**ci**
Below Median Body Fat	29	2.198	1.170	0.217	0.445
Median Body Fat	17	2.188	1.062	0.258	0.546
Above Median Body Fat	6	2.187	0.679	0.277	0.713
Below Median Net Mass	27	2.140	1.162	0.224	0.460
Median Net Mass	23	2.253	1.028	0.214	0.445
Above Median Net Mass	2	2.229	0.438	0.310	3.934
Below Median Lean Mass	22	2.116	1.228	0.262	0.544
Median Lean Mass	23	2.320	0.947	0.198	0.410
Above Median Lean Mass	7	2.019	1.049	0.396	0.970
Below Median Fat Mass	39	2.101	1.094	0.175	0.355
Median Fat Mass	10	2.636	1.052	0.333	0.752
Above Median Fat Mass	3	1.912	0.630	0.364	1.566

c. Inverse Simpson					
**Body Condition**	**N**	**Diversity**	**sd**	**se**	**ci**
Below Median Body Fat	29	8.593	14.828	2.753	5.640
Median Body Fat	17	6.203	5.428	1.316	2.791
Above Median Body Fat	6	5.080	3.659	1.494	3.840
Below Median Net Mass	27	8.443	15.431	2.970	6.104
Median Net Mass	23	6.405	4.932	1.028	2.133
Above Median Net Mass	2	4.918	1.377	0.973	12.367
Below Median Lean Mass	22	8.525	16.903	3.604	7.494
Median Lean Mass	23	6.847	5.433	1.133	2.349
Above Median Lean Mass	7	5.728	3.850	1.455	3.561
Below Median Fat Mass	39	7.470	12.968	2.076	4.204
Median Fat Mass	10	8.139	6.316	1.997	4.518
Above Median Fat Mass	3	4.129	1.678	0.969	4.169

Supplementary Table S9. One-way analysis of variance test for significant differences in brown bear (*Ursus arctos*) gut microbiome alpha diversity between body metrics. No significant differences.

a. Faith's PD					
	**df**	**Sum Sq**	**Mean Sq**	**F value**	**Pr(>F)**
Body Fat	2	35.30	17.650	0.29	0.75
Residuals	49	2987.70	60.970		
Net Mass	2	19.1	9.54	0.156	0.856
Residuals	49	3003.9	61.30		
Lean Mass	2	64	32.23	0.235	0.792
Residuals	49	6728	137.31		
Fat Mass	2	124.2	62.08	1.049	0.358
Residuals	49	2898.9	59.16		

b. Shannon diversity					
	**df**	**Sum Sq**	**Mean Sq**	**F value**	**Pr(>F)**
Body Fat	2	0.00	0.001	0.001	0.999
Residuals	49	58.72	1.198		
Net Mass	2	0.16	0.080	0.067	0.935
Residuals	49	58.56	1.195		
Lean Mass	2	0.72	0.3578	0.302	0.741
Residuals	49	58.01	1.1838		
Fat Mass	2	2.53	1.263	1.101	0.341
Residuals	49	56.20	1.147		

c. Inverse Simpson					
	**df**	**Sum Sq**	**Mean Sq**	**F value**	**Pr(>F)**

Body Fat	2	98	48.94	0.358	0.701
Residuals	49	6695	136.62		
Net Mass	2	64	32.23	0.235	0.792
Residuals	49	6728	137.31		
Lean Mass	2	0.72	0.3578	0.302	0.741
Residuals	49	58.01	1.1838		
Fat Mass	2	38	18.87	0.137	0.872
Residuals	49	6755	137.85		

Supplementary Table S10. W^*_d test results for beta diversity indices comparing brown bears (*Ursus arctos*) with varying body conditions and their GMBs**.** Number of permutations was set to 9999 for all analysis.

a. Weighted

**Body Condition**	**W^*_d stat**	**P value**
Body Fat	0.611	0.923
Net Mass	0.911	0.329
Lean Mass	1.300	0.135
Fat Mass	0.611	0.923

b. Unweighted

**Body Condition**	**W^*_d stat**	**P value**
Body Fat	0.955	0.446
Net Mass	0.774	0.636
Lean Mass	0.985	0.346
Fat Mass	0.955	0.452

Supplementary Table S11. Microbial taxa significantly (p<0.05) enriched in gut microbiomes of brown bears (*Ursus arctos*) with varying health metrics, as determined by Linear discrimination analysis Effect Size analysis.

a. % Body Fat

**Phylum**	**Class**	**Order**	**Family**	**Genus**	**Log LDA**
**Median**					
Cyanobacteria	Melainabacteria				4.273
**Above**					
Firmicutes	Clostridia	Clostridiales	Peptostreptococcaceae		4.303

b. Net Mass

**Phylum**	**Class**	**Order**	**Family**	**Genus**	**Log LDA**
**Median**					
Firmicutes	Clostridia	Clostridiales	Clostridiaceae_1		4.481
Proteobacteria	Gammaproteobacteria	Pasteurellales			4.379

**Above**					
Actinobacteria	Thermoleophilia	Solirubrobacterale	Solirubrobacteraceae	*Solirubrobacter*	3.525

c. Lean Mass

Phylum	Class	Order	Family	Genus	Log LDA
**Below**					
Actinobacteria	Actinobacteria	Propionibacteriales			3.772
Actinobacteria	Actinobacteria	Pseudonocardiales			3.632
Bacteroidetes	Bacteroidia	Flavobacteriales	Flavobacteriaceae	*Flavobacterium*	3.978
**Median**					
Actinobacteria	Actinobacteria	Propionibacteriales	Nocardioidaceae	*Nocardioides*	3.866
Firmicutes	Clostridia	Clostridiales	Clostridiaceae 1	*Clostridium sensu stricto 13*	3.430
**Above**					
Epsilonbacteraeota	Campylobacteria	Campylobacterales	Helicobacteraceae	*Helicobacter*	4.751
Firmicutes	Clostridia	Clostridiales	Lachnospiraceae	*Cellulosilyticum*	3.915

d. Fat Mass

Phylum	Class	Order	Family	Genus	Log LDA
**Median**					
Actinobacteria	Actinobacteria	Micrococcales	Dermacoccaceae		3.266
Acidobacteria	Acidobacteriia	Uncultured eubacterium WD244			2.065
Chlamydiae	Chlamydiae	Chlamydiales			2.224
Chlamydiae	Chlamydiae	Chlamydiales	Parachlamydiaceae	*Candidatus Protochlamydia*	2.149
Dependentiae	Babeliae				2.923
Dependentiae					2.952
Firmicutes	Clostridia	Clostridiales	Peptococcaceae		2.581
Firmicutes	Bacilli	Bacillales	XI	*Gemella*	2.426
Firmicutes	Bacilli	Bacillales	XI		2.445
Firmicutes	Bacilli	Lactobacillales	Streptococcaceae	*Lactococcus*	2.027
Firmicutes	Uncultured				2.223
Firmicutes	Uncultured				2.159
Firmicutes	Uncultured				2.085
Firmicutes	Uncultured				2.174
Planctomycete					2.072
**Above**					
Actinobacteria	Coriobacteriia	Coriobacteriales	Atopobiaceae	*Olsenella*	2.645
Chloroflexi	Ktedonobacteria	Ktedonobacterales	Ktedonobacteraceae		2.155

Elusimicrobia			2.002
Proteobacteria	Gammaproteobacteria	R7C24	2.357

Supplementary Table S12. Spearman's non-parametric correlation analysis between **a)** major phyla (≥1% relative abundance) identified in brown bear (*Ursus arctos*) gut microbiomes and body condition measurements, **b)** dominant genera (≥10% mean abundance) and body condition measurements, and **c)** alpha diversity indices and body condition measurements.

a. Major Phyla

Phylum	Body Metric	S	P value	Rho
Actinobacteria	Body fat (%)	18291	0.649	0.067
	Net body mass (kg)	20784	0.989	0.002
	Lean mass (kg)	19684	0.977	-0.004
	Fat mass (kg)	18456	0.690	0.058
Bacteroidetes	Body fat (%)	20828	0.669	-0.063
	Net body mass (kg)	21687	0.775	-0.041
	Lean mass (kg)	19899	0.917	-0.015
	Fat mass (kg)	20527	0.747	-0.047
Epsilonbacteraeota	Body fat (%)	20908	0.649	-0.067
	Net body mass (kg)	25476	0.119	-0.223
	Lean mass (kg)	22159	0.371	-0.131
	Fat mass (kg)	21065	0.610	-0.075
Firmicutes	Body fat (%)	22476	0.775	0.041
	Net body mass (kg)	23392	0.686	0.057
	Lean mass (kg)	21540	0.571	0.081
	Fat mass (kg)	21918	0.650	0.064
Proteobacteria	Body fat (%)	23341	0.980	0.004
	Net body mass (kg)	26653	0.596	-0.075
	Lean mass (kg)	26732	0.318	-0.141
	Fat mass (kg)	24932	0.651	-0.064
Tenericutes	Body fat (%)	2398	0.843	-0.043
	Net body mass (kg)	2544	0.919	0.022
	Lean mass (kg)	2111	0.702	0.082
	Fat mass (kg)	2279	0.966	-0.009

b. Dominant Genera

Genus	Body Metric	S	P value	Rho
Escherichia-Shigella	Body fat (%)	24756	0.689	-0.057
	Net body mass (kg)	29172	0.207	-0.176
	Lean mass (kg)	27841	0.181	-0.188
	Fat mass (kg)	26575	0.342	-0.134
Streptococcus	Body fat (%)	16977	0.359	0.134
	Net body mass (kg)	20781	0.988	0.002
	Lean mass (kg)	20729	0.694	-0.058
	Fat mass (kg)	17166	0.395	0.124

Turicibacter	Body fat (%)	15592	0.523	-0.099
	Net body mass (kg)	16374	0.608	-0.079
	Lean mass (kg)	14475	0.897	-0.020
	Fat mass (kg)	15733	0.482	-0.109
Clostridium sensu stricto 1	Body fat (%)	20880	0.656	-0.065
	Net body mass (kg)	20398	0.888	0.021
	Lean mass (kg)	17942	0.564	0.085
	Fat mass (kg)	20651	0.714	-0.054
Helicobacter	Body fat (%)	19573	0.674	- 0.062
	Net body mass (kg)	24806	0.065	- 0.266
	Lean mass (kg)	21729	0.223	- 0.179
	Fat mass (kg)	20075	0.545	- 0.090

c. Alpha Diversity

**Diversity Index**	**Body Metric**	**S**	**P value**	**Rho**
Faith's PD	Body fat (%)	21977	0.662	0.062
	Net body mass (kg)	22547	0.7917	0.038
	Lean mass (kg)	22702	0.828	0.031
	Fat mass (kg)	21409	0.544	0.086
Shannon	Body fat (%)	21187	0.500	0.096
	Net body mass (kg)	20245	0.337	0.136
	Lean mass (kg)	19458	0.230	0.169
	Fat mass (kg)	19631	0.251	0.162
Inverse Simpson's	Body fat (%)	22467	0.773	0.041
	Net body mass (kg)	19686	0.258	0.160
	Lean mass (kg)	18714	0.153	0.201
	Fat mass (kg)	20242	0.337	0.136

Supplementary Table S13. Metadata for each brown bear (*Ursus arctos*) sampled during 2015-2017 National Park Service research activities.

Sample ID	ASVs	Initial diversity (non-normalized)	Retained diversity (normalized)	% Retained diversity (normalized)	Body Fat (%)	Net Body Mass (kg)	Lean Mass (kg)	Fat Mass (kg)
G14006	22339	219	216	98.630	13.530	76.854	87.252	10.398
G14011	31490	185	173	93.514	6.951	84.991	79.083	5.908
G14020	49920	124	99	79.839	12.683	98.101	85.659	12.442
G14026	39502	44	39	88.636	4.673	86.799	82.743	4.056
G14030	35343	24	22	91.667	4.155	94.937	90.992	3.944
G14032	28538	108	107	99.074	7.532	79.114	73.155	5.959
G15001	33444	56	50	89.286	3.000	132.459	129.993	2.467
G15003	31898	74	69	93.243	3.000	79.566	78.523	1.043
G15006	8319	27	27	100	11.160	81.826	72.695	9.132
G15009	29794	34	32	94.118	8.701	89.060	81.310	7.749
G16001	24938	53	50	94.34	17.154	94.937	78.651	16.286
G16003	47613	39	32	82.051	13.839	90.416	77.903	12.513
G16005	30105	41	36	87.805	3.000	133.363	131.221	2.1429
G16008	12580	37	37	100	15.332	100.814	85.357	15.457
G16009	38494	141	122	86.525	33.735	247.740	164.164	83.575
K106	27281	58	48	82.759	14.8	180.800	154.100	26.700
K116	28691	156	141	90.385	12.000	145.100	127.700	17.400
K117	36917	62	59	95.161	9.200	157.800	143.200	14.500
K16A1	47070	101	94	93.069	11.400	87.300	77.300	9.900
K16A2	19156	275	267	97.091	3.600	100.800	97.100	3.700
K16A3	13014	215	215	100	32.300	142.400	96.400	46.100
K37	23158	108	106	98.148	22.200	179.000	139.200	39.800
K46	27338	186	150	80.645	17.000	220.200	182.700	37.500

K55	50743	53	44	83.019	16.900	150.100	124.800	25.300
K56A1	68370	73	59	80.822	24.600	197.600	149.000	48.500
K56A2	14684	77	77	100	18.400	220.600	179.900	40.700
K56A3	19635	28	28	100	37.300	302.900	190.000	112.900
K57B1	16611	82	82	100	17.500	117.100	96.600	20.500
K57B2	46593	104	80	76.923	6.800	161.400	150.400	11.000
K57B3	43852	51	47	92.157	20.700	167.700	133.000	34.700
K66	33904	328	323	98.476	8.500	124.300	113.700	10.600
K67		Dropped			5.100	169.500	161.000	8.600
K76A1	51412	375	320	85.333	14.700	157.800	134.600	23.200
K76A2	17191	101	99	98.020	14.900	170.000	144.600	25.300
K76A3	51655	101	63	62.376	39.00	214.300	130.800	83.500
K76B1	52626	92	67	72.826	7.900	146.900	135.400	11.500
K76B2	59738	50	40	80	21.700	188.500	147.700	40.800
K86		Dropped			15.100	172.200	146.300	25.900
K87B1	40277	55	49	89.091	18.700	119.800	97.400	22.400
K87B2	4087	180	180	100	25.100	154.200	115.500	38.700
K95A2	23082	32	29	90.625	3.000	202.100	198.600	3.500
K96	50225	98	88	89.796	22.500	222.400	172.000	50.000
K97B1	35732	534	526	98.502	8.600	120.300	109.900	10.400
K97B2	13660	124	124	100	20.600	168.200	133.500	34.700
K97B3	63873	211	157	74.408	31.700	172.200	117.700	54.600
L401	26880	107	87	81.308	9.355	160.940	145.884	15.056
L405	65660	76	52	68.421	13.817	98.101	84.547	13.555
L412	49341	47	27	57.447	10.542	121.157	108.385	12.773
L415	7241	58	58	100	8.657	165.913	151.550	14.364
L421A	57236	56	33	58.929	13.192	198.463	172.282	26.181
L423	51326	55	31	56.364	19.674	96.745	77.7118	19.033
L521	14106	378	377	99.735	22.953	192.586	148.382	44.204

L522	46327	210	171	81.429	27.142	165.009	120.28	44.787
L601	60402	72	41	56.944	21.728	155.967	122.079101	33.888
